# Evolution of the ancestral mammalian karyotype and syntenic regions

**DOI:** 10.1073/pnas.2209139119

**Published:** 2022-09-26

**Authors:** Joana Damas, Marco Corbo, Jaebum Kim, Jason Turner-Maier, Marta Farré, Denis M. Larkin, Oliver A. Ryder, Cynthia Steiner, Marlys L. Houck, Shaune Hall, Lily Shiue, Stephen Thomas, Thomas Swale, Mark Daly, Jonas Korlach, Marcela Uliano-Silva, Camila J. Mazzoni, Bruce W. Birren, Diane P. Genereux, Jeremy Johnson, Kerstin Lindblad-Toh, Elinor K. Karlsson, Martin T. Nweeia, Rebecca N. Johnson, Zoonomia Consortium, Harris A. Lewin

**Affiliations:** ^a^The Genome Center, University of California, Davis, CA 95616;; ^b^Department of Biomedical Science and Engineering, Konkuk University, Seoul 05029, South Korea;; ^c^Broad Institute of Massachusetts Institute of Technology and Harvard, Cambridge, MA 02142;; ^d^School of Biosciences, University of Kent, Canterbury CT2 7NJ, United Kingdom;; ^e^The Royal Veterinary College, University of London, London NW1 0TU, United Kingdom;; ^f^Conservation Genetics, San Diego Zoo Wildlife Alliance, Escondido, CA 92027;; ^g^Department of Evolution, Behavior, and Ecology, Division of Biology, University of California San Diego, La Jolla, CA 92093;; ^h^Conservation Science Wildlife Health, San Diego Zoo Wildlife Alliance, Escondido, CA 92027;; ^i^Beckman Center for Conservation Research, San Diego Zoo Wildlife Alliance, Escondido, CA 92027;; ^j^Dovetail Genomics, LLC, Scotts Valley, CA 95066;; ^k^Pacific Biosciences, Menlo Park, CA 94025;; ^l^Wellcome Sanger Institute, Cambridgeshire CB10 1SA, United Kingdom;; ^m^Berlin Center for Genomics in Biodiversity Research, D-14195 Berlin, Germany;; ^n^Evolutionary Genetics Department, Leibniz Institut für Zoo- und Wildtierforschung, 10315 Berlin, Germany;; ^o^Science for Life Laboratory, Department of Medical Biochemistry and Microbiology, Uppsala University, Uppsala 751 23, Sweden;; ^p^Bioinformatics and Integrative Biology, University of Massachusetts Medical School, Worcester, MA 01655;; ^q^Program in Molecular Medicine, University of Massachusetts Medical School, Worcester, MA 01655;; ^r^Department of Restorative Dentistry and Biomaterials Sciences, Harvard School of Dental Medicine, Boston, MA 02115;; ^s^Department of Comprehensive Care, Case Western Reserve University School of Dental Medicine, Cleveland, OH 44106;; ^t^Marine Mammal Program, Department of Vertebrate Zoology, Smithsonian Institution, Washington, DC 20002;; ^u^Australian Museum Research Institute, Australian Museum, Sydney, NSW 2010, Australia;; ^v^School of Life and Environmental Sciences, Faculty of Science, University of Sydney, Sydney, NSW 2006, Australia;; ^w^Department of Evolution and Ecology, University of California, Davis, CA 95616;; ^x^John Muir Institute for the Environment, University of California, Davis, CA 95616

**Keywords:** chromosome evolution, mammals, synteny conservation, ancestral genome reconstruction, topologically associating domains

## Abstract

Decrypting the rearrangements that drive mammalian chromosome evolution is critical to understanding the molecular bases of speciation, adaptation, and disease susceptibility. Using 8 scaffolded and 26 chromosome-scale genome assemblies representing 23/26 mammal orders, we computationally reconstructed ancestral karyotypes and syntenic relationships at 16 nodes along the mammalian phylogeny. Three different reference genomes (human, sloth, and cattle) representing phylogenetically distinct mammalian superorders were used to assess reference bias in the reconstructed ancestral karyotypes and to expand the number of clades with reconstructed genomes. The mammalian ancestor likely had 19 pairs of autosomes, with nine of the smallest chromosomes shared with the common ancestor of all amniotes (three still conserved in extant mammals), demonstrating a striking conservation of synteny for ∼320 My of vertebrate evolution. The numbers and types of chromosome rearrangements were classified for transitions between the ancestral mammalian karyotype, descendent ancestors, and extant species. For example, 94 inversions, 16 fissions, and 14 fusions that occurred over 53 My differentiated the therian from the descendent eutherian ancestor. The highest breakpoint rate was observed between the mammalian and therian ancestors (3.9 breakpoints/My). Reconstructed mammalian ancestor chromosomes were found to have distinct evolutionary histories reflected in their rates and types of rearrangements. The distributions of genes, repetitive elements, topologically associating domains, and actively transcribed regions in multispecies homologous synteny blocks and evolutionary breakpoint regions indicate that purifying selection acted over millions of years of vertebrate evolution to maintain syntenic relationships of developmentally important genes and regulatory landscapes of gene-dense chromosomes.

## Significance

Computational reconstruction of ancestral mammalian karyotypes revealed a comprehensive picture of the chromosome rearrangements that occurred over the evolutionary history of mammals. Ancient gene order, in some cases extending to full chromosomes, was found conserved for more than 300 My, demonstrating strong evolutionary constraint against rearrangements in some regions. Conserved segments of chromosomes are enriched for genes that control developmental processes. Therefore, Darwinian selection likely maintains ancient gene combinations while allowing for genomic innovations within or near chromosomal sites that break and rearrange over evolutionary time. The revealed relationship between the three-dimensional structure of chromosomes and the evolutionary stability of chromosome segments provides additional insights into the mechanisms of chromosome evolution and diseases associated with genome rearrangements.

Resolving karyotypes and syntenic relationships in common ancestors along phylogenetic lineages facilitates the identification and dating of chromosomal rearrangements that have led to the organization of extant genomes, ultimately shedding light on species biology and evolutionary history (1). Earlier studies of mammalian chromosome evolution focused on placental (2–7) or marsupial mammals (8, 9). Limitations of chromosome painting and the highly fragmented nature of first-generation genome assemblies impeded the reconstruction of ancestral karyotypes older than 100 My in the eutherian lineage (10). Additionally, a dearth of chromosome-scale genome assemblies for monotremes and marsupials has delayed sequence-based reconstruction of the ancestral mammalian karyotype, deferring a comprehensive study of the evolution of mammalian chromosomes.

Despite these limitations, studies of chromosome evolution led to important insights into the mechanisms that drive chromosome rearrangements and their role in adaptation and speciation (reviewed in ref. 1). These insights have informed the theory of chromosomal speciation, which posits that chromosome rearrangements promote reproductive isolation by suppressing recombination in rearranged regions and subsequent accumulation of genetic incompatibilities (11). Examples of chromosomal speciation can be found in multiple mammalian species, including rock wallaby (12) and *Rhogeessa* bats (13). Rearranged regions are also associated with the accumulation of genes linked to adaptive phenotypes and are responsible for a wide variety of disease phenotypes, including leukemias and lymphomas (14).

One of the most challenging problems in studying chromosome evolution in vertebrates is demonstrating direct cause and effect relationships between chromosome rearrangements and phenotypes. Much of what we know about the mechanisms governing chromosome evolution is derived from empirical observations of karyotypic differences between species, the characterization of evolutionary breakpoint regions (EBRs) and homologous synteny blocks (HSBs), and their association with different sequence features (3, 15–22). The distribution of EBRs in the genome is nonrandom, which together with the observed overlap between EBRs and cancer breakpoints and the reuse of breakpoints during evolution, suggest that chromosomes have fragile sites with a higher likelihood for breakage (5, 15, 23–28). Multiple lines of evidence support this hypothesis, including the association of EBRs with repetitive sequences (15, 17, 29, 30). The high gene density observed in EBRs could also explain their greater propensity for double-strand breaks because transcriptionally active open chromatin is more susceptible to DNA damage, as shown for postmeiotic cells (31). However, these reasons cannot solely explain the distribution of evolutionary breakpoints within a genome. An additional possibility is that selection acts to limit disruptions of syntenic segments that include developmentally important genes and regulatory networks. The higher numbers of developmental and housekeeping genes and conserved noncoding elements in HSBs (15, 18, 21, 32) support this hypothesis, as do the more recent observations that EBRs rarely disrupt topologically associating domains (TADs) (33, 34). Despite these advances, many questions concerning the evolutionary mechanisms of chromosome evolution are still unanswered. For example, it has not been shown conclusively that HSBs remain as unbroken gene clusters because of selective constraints rather than by chance or that gene networks are rewired because of chromosome rearrangements.

The combined efforts of large-scale genome sequencing projects (35–37) have provided an increasing number of high-quality, chromosome-scale genome assemblies for mammals that can be used to address these issues. Herein, we used chromosome-scale genome assemblies representing 23/26 mammal orders to computationally reconstruct ancestral karyotypes and syntenic relationships at 16 nodes along the mammalian phylogeny, revealing the rearrangement patterns of ancestral mammalian chromosomes during ∼180 My of evolution. Analysis of sequence features of EBRs and HSBs provided insights into the mechanisms leading to synteny conservation or chromosome breakage in genome evolution.

## Results

### Reconstruction of 16 Ancestral Karyotypes along the Mammalian Phylogeny.

**Fig. 1.**
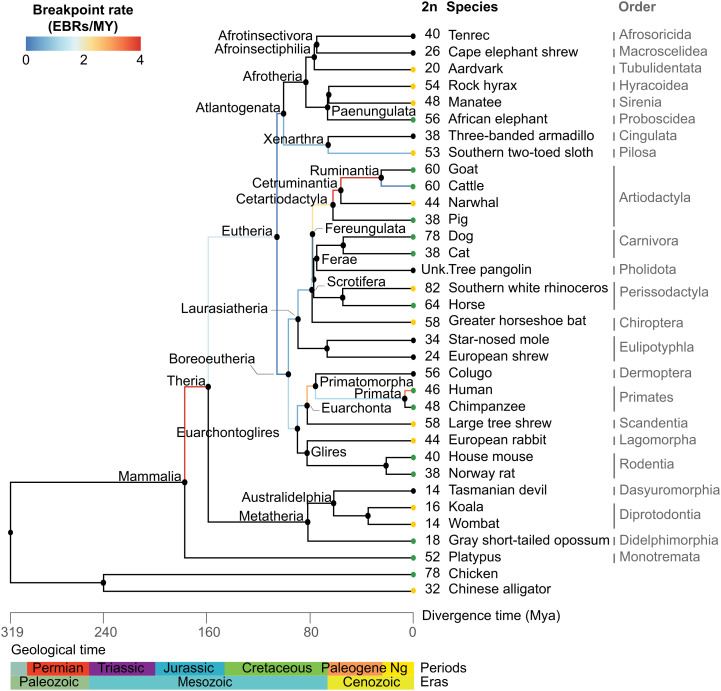
Phylogenetic tree of descendant species and reconstructed ancestors. The branch color represents breakpoint rates in RACFs (breakpoints per million years). Black branches represent nondetermined breakpoint rates. Tip colors depict assembly contiguity: black, scaffold-level genome assembly; green, chromosome-level genome assembly; yellow, chromosome-scale scaffold-level genome assembly. Numbers next to species names indicate diploid chromosome number (if known).

The sequences of 32 extant mammals (Dataset S1), representing all 19 eutherian, 3 marsupial, and the monotreme orders, were used to reconstruct ancestral karyotypes at 16 nodes of the mammalian phylogeny, including the common ancestor of all mammals (Fig. 1 and *SI Appendix*, Table S1). We chose the human, cattle, and Southern two-toed sloth (“sloth” hereafter) genome assemblies as references because they are members of three mammalian lineages (Euarchonta, Laurasiatheria, and Xenarthra, respectively) and have high assembly quality and contiguity relative to other representatives of their clades. The average pairwise whole-genome alignment coverages within each lineage were >93% (Dataset S2). We used DESCHRAMBLER (3) to generate reconstructed ancestral chromosome fragments (RACFs) for each of 16 mammalian ancestors at 300-kbp syntenic fragment (SF) resolution. These RACFs were then manually curated to obtain ancestral chromosomes (*SI Appendix*). The chromosome assemblies for every reconstructed ancestor are shown in Datasets S3–S5. The combined length of RACFs assigned to each ancestor’s chromosomes represents >99% of the total of each reconstructed ancestor’s genome length (*SI Appendix*, Table S1). The reconstructed mammalian ancestor karyotype includes ∼87% of the genome sequence of each of the three references (*SI Appendix*, Table S1) and contains ≥83% of complete mammalian benchmarking universal single-copy orthologs [BUSCOs (38)] (*SI Appendix*, Fig. S1). For all reconstructions, except the ruminant ancestor, more recent ancestors had sequentially higher reference genome coverage (88 to 98%) (*SI Appendix*, Table S1) and more complete mammalian BUSCOs (88 to 96%) (*SI Appendix*, Fig. S1).

### Assessment of Reference Bias in the Reconstructed Ancestral Karyotypes.

We compared the mammalian, therian, eutherian, and boreoeutherian ancestor chromosomes reconstructed using the three reference genomes. The human genome-based reconstructions at the different ancestral nodes were generally in higher agreement with those obtained using sloth as the reference genome than with the cattle genome-based reconstructions (*SI Appendix*, Fig. S2–S5 and Tables S2–S4). This was likely due to differences in target species genome coverage (Dataset S6), particularly for the early-diverging platypus, which was important for resolving the chromosome organization of the mammalian ancestor. Even so, >90 and >79% of the total lengths of the reconstructions were in agreement for the human–sloth and human–cattle reconstruction comparisons, respectively (*SI Appendix*, Table S4). The cumulative length of the syntenic segments with structural disagreements between the different reconstructions was much lower for more recent ancestors, with <0.5% inconsistency for the eutherian and boreoeutherian ancestors (*SI Appendix*, Table S4). The highest BUSCO scores were obtained for the ancestral chromosomes reconstructed using the human genome sequence as the reference (*SI Appendix*, Fig. S1). In addition, the human genome-based reconstruction of the mammalian ancestor recovered the most complete mammalian BUSCOs shared between the human and platypus genomes (99%) (*SI Appendix*, Table S5). Because the human genome-based reconstructions of the mammalian, therian, eutherian, and boreoeutherian chromosome organization were the most comprehensive, we chose them for subsequent analyses of chromosome evolution presented below (*SI Appendix* has visualizations and statistics for the sloth and cattle genome-based reconstructions).

### Evolution of the Mammalian Ancestor Chromosomes in the Human, Cattle, and Sloth Lineages.

**Fig. 2.**
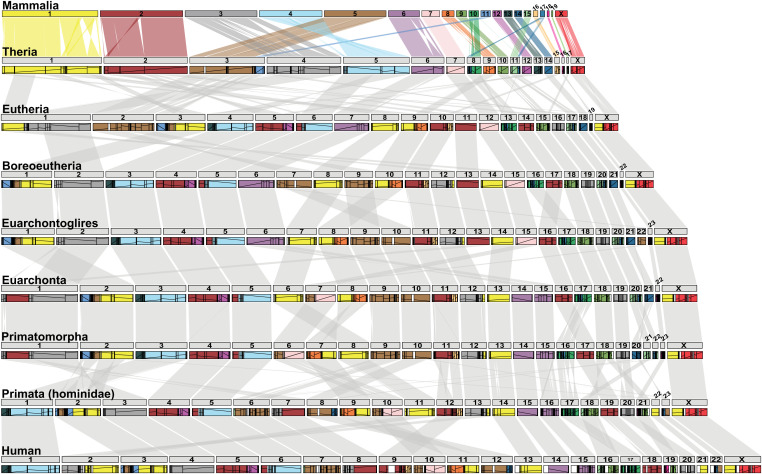
Evolution of MAMs in the lineage leading to humans. MAMs are distinguished by the colors at the top of the diagram. Colored blocks for every other ancestor and human depict the orthology to MAMs. Lines within colored blocks represent block orientation compared with the MAMs, with positive and negative slopes portraying the same or different orientations, respectively. Gray ribbons depict the orthology of each ancestor to its phylogenetically closest ancestors or species. An orthology map for each pairwise comparison is presented in Dataset S12.

The reconstructed mammalian ancestor karyotype has 19 autosomes plus X, except for the cattle genome-based reconstruction, which has two fewer chromosomes and the lowest total reconstruction length (Fig. 2, Datasets S4–S6, and *SI Appendix*, Figs. S6 and S7 and Table S1). The X chromosome was assigned according to its orthology to the X chromosome of therian mammals. The evolutionary history of the Y chromosome could not be established because only 9 of 32 mammal species, from four orders, were represented by males (Dataset S1). The differences between the mammalian and therian (*n* = 17 + X) ancestors’ chromosomes resulted from 96 chromosomal rearrangements over 18 My (Fig. 2 and *SI Appendix*, Figs. S6 and S7 and Table S6). The mammalian and therian reconstructions recovered all previously reported ancestral syntenies and facilitated the discovery of >10 ancestral syntenies (*SI Appendix*, Table S7). Two of these were previously classified as xenarthran specific, represented by the association of human chromosome (HSA) 8p with part of HSA2pq and of HSA10p with part of HSA7pq (39). Another was previously reported as bat specific, represented by the association of part of HSA1pq with part of HSA6pq (40). We found additional support for these syntenies in the platypus and marsupial genomes, suggesting that they are ancestral to all mammals and not clade specific. The eutherian ancestor (*n* = 19 + X) is the most recent common ancestor of the three lineages represented by the reference genomes. Its karyotype evolved from that of the therian ancestor as a result of 124 chromosomal rearrangements over 53 My. The eutherian ancestral karyotype is distinguished from the descendant boreoeutherian ancestral karyotype (*n* = 22 + X; the most recent common ancestor to the human and cattle lineages) by four chromosomal rearrangements that occurred over 9 My (Fig. 2 and *SI Appendix*, Figs. S6 and S7 and Table S6). We detected one unreported ancestral synteny, represented by the association of all of HSA5 and part of HSA1q (*SI Appendix*, Table S7), which is substantiated by all extant eutherian genomes except for primates and species with fragmented (scaffold-level) genome assemblies.

In the sloth lineage, we reconstructed two additional ancestral karyotypes, Atlantogenata and Xenarthra. The reconstructed atlantogenatan ancestral karyotype has 19 autosomes plus X, which closely resemble the antecedent eutherian ancestral karyotype except for four inversions that occurred over 5 My (*SI Appendix*, Fig. S6 and Table S6). The xenarthran ancestral karyotype (*n* = 24 + X) evolved from that of the atlantogenatan ancestor by 36 chromosomal rearrangements over 35 My. The descendent sloth genome (*n* = 26 + X) differs from the xenarthran ancestor by 51 chromosomal rearrangements that occurred over 66 My (*SI Appendix*, Fig. S6 and Table S6). All reported xenarthran ancestral syntenies (41) were recovered, and four others were identified (*SI Appendix*, Table S7). Two are retained from the atlantogenatan ancestor and are represented by the association of HSA4 with HSA8p and part of HSA2p and of HSA10p with parts of HSA12q and HSA22. The other two are xenarthran specific, represented by the association of HSA1 with part of HSA6q and parts of HSA5q and HSA6q.

In the lineage leading to cattle, karyotypes were reconstructed at six other ancestral nodes descending from the boreoeutherian ancestor (Laurasiatheria, Scrotifera, Fereungulata, Cetartiodactyla, Cetruminantia, and Ruminantia). The laurasiatherian ancestor karyotype (*n* = 23 + X) differs from that of the boreoeutherian ancestor by 10 chromosomal rearrangements that occurred over 7 My (*SI Appendix*, Fig. S7 and Table S6). All ancestral syntenies found in the laurasiatherian ancestor were retained in the descendent scrotiferan ancestral karyotype (*n* = 24 + X) (*SI Appendix*, Fig. S7 and Table S6), but the ancestral synteny represented by the association of parts of HSA8p and HSA4pq is found in two distinct chromosomes because of a chromosomal fission that occurred over the 11 My separating these two ancestors (*SI Appendix*, Table S7). We also reconstructed the fereungulatan ancestor chromosomes (*n* = 23 + X), which differ from those of the antecedent scrotiferan ancestor by five rearrangements that occurred over 1 My (*SI Appendix*, Fig. S7 and Table S6). For the fereungulatan ancestor, we discovered four ancestral syntenies (*SI Appendix*, Table S7). Two are retained from the scrotiferan ancestor, represented by the association of parts of HSA4q and HSA8p and HSA5 with part of HSA1q. The other two are Ferungulata specific, represented by the association of HSA10q with part of HSA1q, and HSA18 with parts of HSA12q and HSA22. The cetartiodactyl ancestor karyotype (*n* = 24 + X) evolved from that of the fereungulatan ancestor by five chromosomal rearrangements over 16 My and was succeeded by the cetruminant ancestor, which was found to share the same chromosome number (*n* = 24 + X) but is differentiated by 23 rearrangements that occurred over 6 My (*SI Appendix*, Fig. S7 and Table S6). These rearrangements led to the appearance of two ancestral syntenies, represented by the associations of HSA20 and HSA16p with part of HSA7pq and parts of HSA15q with HSA2q (*SI Appendix*, Table S7). The reconstructed ruminant (Bovidae) ancestor karyotype (*n* = 29 + X) evolved from the cetruminant ancestor as a result of 107 chromosomal rearrangements over 31 My, and while it has the same chromosome number as cattle, the genomes differ by six inversions that happened over 25 My (*SI Appendix*, Fig. S7 and Table S6).

We reconstructed four ancestral karyotypes in the lineage leading from the boreoeutherian ancestor to humans (Euarchontoglires, Euarchonta, Primatomorpha, and Primata). The Euarchontoglires ancestral karyotype (*n* = 23 + X) evolved from that of the boreoeutherian ancestor by five chromosomal rearrangements over 7 My (Fig. 2 and *SI Appendix*, Table S6), retaining all of its ancestral syntenies (*SI Appendix*, Table S7). The ancestral karyotype of Euarchonta (*n* = 22 + X) differs from its antecedent, the Euarchontoglires ancestor, by 12 chromosomal rearrangements over 8 My and its descendant, the primatomorphan ancestor, by 27 rearrangements that occurred over 6 My (Fig. 2 and *SI Appendix*, Table S6). The most recent ancestor reconstructed for the human lineage was that of primates (Hominidae; *n* = 23 + X), which differs from the antecedent primatomorphan ancestor by 81 chromosomal rearrangements that occurred over 69 My and differs from the human genome by 16 rearrangements that occurred over 7 My (Fig. 2 and *SI Appendix*, Table S6).

### Rates of Chromosome Evolution during Mammalian Evolution.

We identified 323, 262, and 257 EBRs that occurred along the lineage from the mammalian ancestor to the human, sloth, and cattle genomes, respectively (Datasets S7–S9 and *SI Appendix*, Tables S6 and S8). Breakpoint rates varied remarkably during the ∼180 My of mammalian evolution, with the highest and lowest rates being ±2× the lineage average (∼2 breakpoints/My) (Fig. 1 and *SI Appendix*, Table S6). The eutherian to boreoeutherian branch had the lowest observed breakpoint rate (<0.2 breakpoints/My), which is significantly lower than the lineage average (false discovery rate [FDR] *P* < 0.05). The highest breakpoint rate was observed in the branch from the mammalian to the therian ancestor (3.9 breakpoints/My; FDR *P* < 0.05). In the human lineage, the branch from the primate ancestor to human also had a breakpoint rate higher than the average (3.3 breakpoints/My; FDR *P* < 0.05). In the cattle lineage, the highest breakpoint rates were observed in the cetartiodactyl to cetruminant and cetruminant to ruminant ancestors (3.8 and 3.6 breakpoints/My, respectively; FDR *P* < 0.05). Differences in breakpoint rates on each branch from the mammalian ancestor to sloth were not significant relative to the sloth lineage average.

We also analyzed the distribution of the number and types of chromosomal rearrangements that occurred in the human, cattle, and sloth lineages. Inversions were most frequent, accounting for 76 to 85% of all rearrangements identified for each lineage (Fig. 2 and *SI Appendix*, Figs. S6 and S7 and Table S6). More than half of the inversions happened along the lineage from the mammalian to the eutherian ancestor (59%; *n* = 184) in the human genome-based reconstructions. The highest number of interchromosomal rearrangements (i.e., fissions and fusions) was observed on the branch from the therian to the eutherian ancestor (*n* = 30) (Fig. 2 and *SI Appendix*, Table S6). Similar rearrangement frequencies were obtained using the sloth genome-based reconstructions (*SI Appendix*, Fig. S6 and Table S6). In the cattle lineage, both the highest number of inversions and interchromosomal rearrangements were detected along the lineage from the cetruminant to the ruminant ancestor (*n* = 87 and 19, respectively) (*SI Appendix*, Fig. S7 and Table S6).

### Evolutionary History of Mammalian Ancestor Chromosomes.

**Fig. 3.**
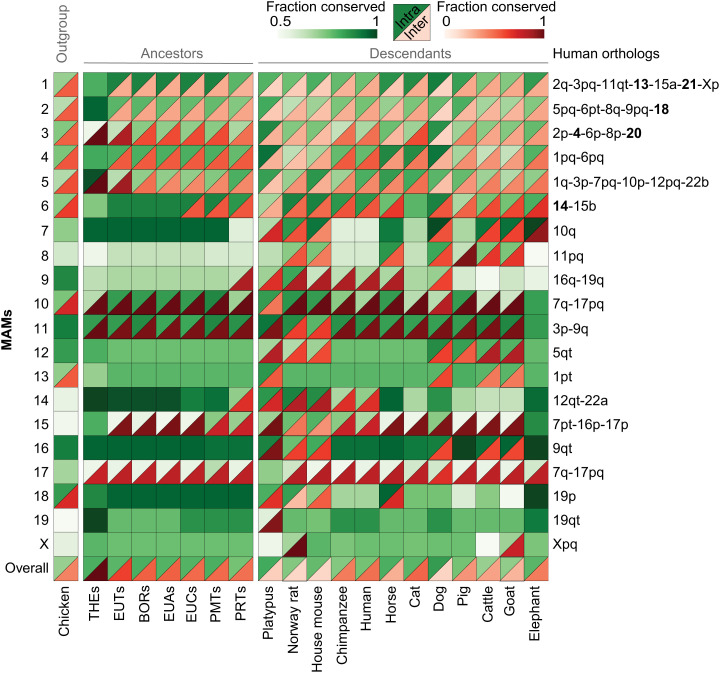
Visualization of the evolutionary history of reconstructed mammalian chromosomes based on the human lineage. Solid green squares indicate mammalian chromosomes maintained as a single synteny block (either as a single chromosome or fused with another MAM), with shades of the color indicating the fraction of the chromosome affected by intrachromosomal rearrangements (the lightest shade is most affected). Split blocks demarcate mammalian chromosomes affected by interchromosomal rearrangements. Upper (green) triangles show the fraction of the chromosome affected by intrachromosomal rearrangements, and lower (red) triangles show the fraction affected by interchromosomal rearrangements. Syntenic relationships of each MAM to the human genome are given at the right of the diagram. MAMX appears split in goat because its X chromosome is assembled as two separate fragments. BOR, boreoeutherian ancestor chromosome; EUA, Euarchontoglires ancestor chromosome; EUC, Euarchonta ancestor chromosome; EUT, eutherian ancestor chromosome; PMT; Primatomorpha ancestor chromosome; PRT, primates (Hominidae) ancestor chromosome; THE, therian ancestor chromosome.

The evolutionary history of mammalian ancestor chromosomes (MAMs) varied considerably depending on their size. Larger MAMs (>100 Mbp; MAM1 to MAM6) were more frequently involved in chromosomal fissions than their smaller counterparts (<100 Mbp; MAM7 to MAM19 and MAMX) (Fig. 3; *SI Appendix*, Figs. S8 and S9 show sloth and cattle genome-based reconstructions). MAM1 to MAM5 (except MAM2 in koala) are orthologous to at least two chromosomes in each extant species, while all the remaining MAMs were maintained as part of a single chromosome in at least one of them (*SI Appendix*, Fig. S10). The propensity of MAMs to undergo intrachromosomal rearrangements was more uniform, as measured by the fraction of the ancestral chromosome that was conserved (Fig. 3). A notable example is MAM7, which had 95% of its length unaffected by intrachromosomal rearrangements until the primatomorphan ancestor (76 Mya). Conservation of synteny at the chromosome level was observed in some extant species (e.g., house mouse, horse, dog, cattle, and goat) in which >90% of MAM7 is conserved. By comparison, MAM8 was only involved in fissions in rodents and some artiodactyls, including the ruminant ancestor; however, on average, 33% of its length was affected by inversions (Fig. 3).

We observed that 9 of 14 small MAMs have 1:1 orthology to chicken chromosomes (GGA) and reconstructed chromosomes of the avian (18) and amniote ancestors (42) (Table 1). These include MAM7, MAM14, and MAM19, which are remarkably conserved during mammalian evolution. Among the remaining small MAMs, MAM10 and MAM18 are orthologous to two full chicken microchromosomes each (GGA18 and GGA27, and GGA28 and GGA30, respectively). MAM8 is orthologous to part of GGA5. MAM13 is orthologous to fragments of two chicken microchromosomes (GGA21 and GGA23), and while MAM19 is orthologous to a complete chicken microchromosome (GGA32), its state in the avian and amniote ancestors is not known. Some MAMs were maintained as distinct chromosomes or as a contained unit (i.e., whole chromosomes fused to one or more chromosomes without a break in synteny in extant mammal genomes). For example, MAM7 was maintained as a single chromosome in European rabbit, greater horseshoe bat, and rock hyrax, which represent three mammal orders (*SI Appendix*, Fig. S10). MAM13 and MAM14 were maintained as a contained chromosomal unit in >15 extant mammals. Together, these results demonstrate conservation of synteny for ∼320 My of evolution since the common ancestor of all amniotes.

**Table 1.** Orthology of the MAMs to HSAs, GGAs, avian ancestor chromosomes, and amniote ancestor chromosomes

MAM	Length (Mbp)	HSA	GGA	Length in GGA (Mbp)	AVI*	AMN^†^
1	414	2q-3pq-11qt-**13**-15a-**21**-Xp	1q-**7**-**9**-**24**	171	1-**7**-**9**-**24**	2-**7**-**12**-**22**
2	354	5pq-6pt-8q-9pq-**18**	2q-Z	146	2-Z	3-5
3	309	2p-**4**-6p-8p-**20**	3pq-4q-**20**-**22**-Z	115	3-**4**-**20**-**22**-Z	1-**4**-**19**-**21**-5
4	269	1pq-6pq	1q-3q-**8**-21-23-**25**-**26**	98	1-3-**8**-21-23-nd-**26**	2-1-**10**-20-nd-nd-**23**
5	274	1q-3p-7pq-10p-12pq-22b	1p-2pq	121	1-2	**6**-**8**-3
6	133	**14**-15b	5pq-**10**	50	5-**10**	nd-**13**
7	76	10q	**6**	26	**6**	**9**
8	52	11pq	5pq	19	5	nd
9	44	16q-19q	**11**	18	**11**	**14**
10	42	7q-17pq	**18**-**27**	13	**18**-**27**	nd-**24**
11	41	3p-9q	**12**	16	**12**	**15**
12	38	5qt	**13**	15	**13**	nd
13	35	1pt	21-23	10	21-23	20-nd
14	31	12qt-22a	**15**	9	**15**	**17**
15	29	7pt-16p-17p	**14**	10	**14**	**16**
16	20	9qt	**17**	9	**17**	**18**
17	20	7q-17pq	**19**	9	**19**	nd
18	9	19p	**28**-**30**	3	nd-nd	nd-nd
19	6	19qt	**32**	0.12	nd	nd
X	54	Xpq	4p	13	**4A**	**11**

MAMs in bold indicate 1:1 orthology between MAMs, GGAs, avian ancestor chromosomes (AVIs), and/or amniote ancestor chromosomes (AMNs). Bold chromosome numbers in HSA, GGA, AVI, or AMN indicate orthology to a unique MAM. nd, not determined.

^*^AVIs from Damas et al. (18).

^†^AMNs from Uno et al. (42).

### Distribution of Protein-Coding Genes in MAMs, Multispecies HSBs, and EBRs.

To investigate why some reconstructed MAMs are more conserved in evolution than others, all human protein-coding genes were mapped to MAMs (16,777/19,878 mapped), and the density of these genes per MAM was calculated. We observed that small MAMs were more gene dense ($\bar{x}$ = 19 complete genes/Mbp) than larger MAMs ($\bar{x}$ = 6 complete genes/Mbp) (*SI Appendix*, Table S9). The complete genes that mapped to smaller MAMs were, on average, smaller ($\bar{x}$ = 51 kbp) than those that mapped to larger MAMs ($\bar{x}$ = 85 kbp) (*SI Appendix*, Table S9).

We then studied the gene landscapes and functions in mammalian multispecies homologous synteny blocks (msHSBs; i.e., those HSBs found in all mammal species we studied). We defined 1,215 mammalian msHSBs longer than 300 kbp covering ∼1.7 Gbp (55%) of the human genome sequence (Dataset S10 and *SI Appendix*, Table S10). Comparison of msHSB sizes in the 24 chromosome-scale assemblies in our dataset showed that the mean msHSB length across all species closely approximates the mean msHSB length as measured by coordinates in the human genome (1.4 Mbp); however, the platypus genome had a much smaller mean msHSB length (962 kbp) (*SI Appendix*, Fig. S11). Five msHSBs were >10 Mbp in the human genome, which is larger than expected by chance (*P* < 0.05). The longest msHSB is ∼22.1 Mbp and is located on chromosome 2q in humans. This msHSB contains 118 human protein-coding genes, 19 of which (including the *HOXD* gene cluster) are involved in embryonic morphogenesis (FDR *P* = 2.3e-07) (Dataset S11). The ∼12-Mbp msHSB located in HSA8q has four genes associated with embryonic limb morphogenesis (FDR *P* = 4.1e-02) (Dataset S11).

We then analyzed the distributions of the number and length of all human protein-coding genes within msHSBs, EBRs identified in the human lineage, or other regions of the human genome (e.g., human-specific sequences, repeats, and HSBs in some but not all mammal species). Due to limitations on the precise definition of EBRs colocating with human centromeres, centromere-associated EBRs (*n* = 12; length >4 Mbp) were not included in the following analysis. We assessed breakpoint reuse for EBRs of <300 kbp. This size limit was chosen to avoid erroneous reuse breakpoint classification due to EBR chaining (5). We found that 29 EBRs were reused in different lineages (9% of EBRs identified in the human lineage). Most reuse was found in rodents (*n* = 20), bovids (*n* = 9), dog (*n* = 9), and pig (*n* = 6) (Dataset S7).

**Fig. 4.**
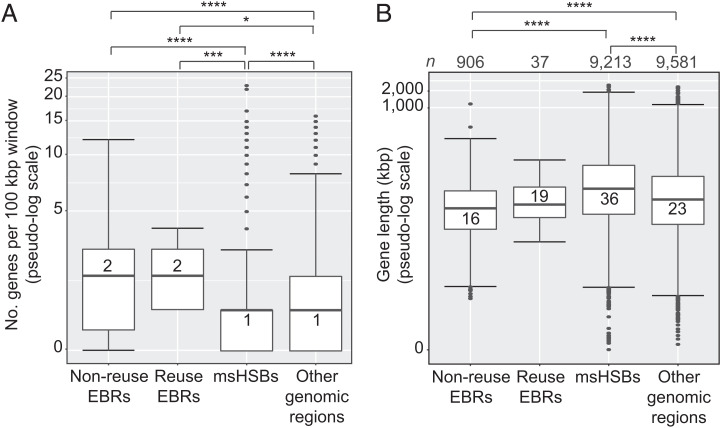
Distribution of human protein-coding genes within EBRs, msHSBs, and other regions of the human genome. (*A*) Number of human protein-coding genes in 100-kbp windows. (*B*) Length distribution of complete human protein-coding genes within nonreuse EBRs, reuse EBRs, msHSBs, and other regions of the human genome. Numbers of complete human protein-coding genes in each category are given at the top of the diagram. (*B*) Numbers within the box plots are group medians. Asterisks depict Bonferroni-corrected *P* values. Only significant comparisons are shown. *P < 0.05; ***P ≤ 0.001; ****P ≤ 0.0001.

Reuse and nonreuse EBRs contained a significantly higher median density of human protein-coding genes (2 genes/100 kbp) than msHSBs or other regions of the genome (1 gene/100 kbp) (Fig. 4*A*). However, complete human genes, including introns, within EBRs have a lower median size (19 kbp) than those in msHSBs and other regions of the genome (36 and 23 kbp, respectively) (Fig. 4*B*). Longer genes in msHSBs were also observed for all other species examined (*SI Appendix*, Table S11). The genes within msHSBs were enriched for functions related to the Gene Ontology (GO) functional terms for biological processes primarily related to anatomical and central nervous system development (Dataset S11 and *SI Appendix*, Fig. S12*A*). Genes within EBRs were enriched for functions primarily related to sensory perception and regulation of transcription (Dataset S11 and *SI Appendix*, Fig. S12*B*).

### Distribution of Repetitive Sequences in msHSBs and EBRs.

**Fig. 5.**
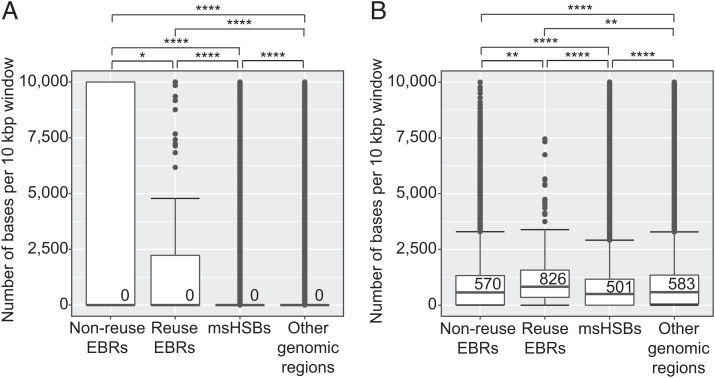
Distribution of repetitive sequences within EBRs, msHSBs, and other regions of the human genome. (*A*) Number of bases within segmental duplications in 10-kbp windows. (*B*) Number of bases within LTRs in 10-kbp windows. Numbers within the box plots are group medians. Asterisks depict Bonferroni-corrected *P* values. Only significant comparisons are shown. Results for other repeat classes and subclasses are shown in *SI Appendix*, Figs. S13 and S14, respectively. *P < 0.05; **P ≤ 0.01; ****P ≤ 0.0001.

The distribution of repetitive sequences within msHSBs and reuse and nonreuse EBRs identified in the human lineage were analyzed using the human genome as a reference. The density of each genomic feature was calculated as the number of bases in 10-kbp windows within the target feature. Both reuse and nonreuse EBRs were found to have a significantly higher density of repeats (all types), segmental duplications, short interspersed nuclear elements (SINEs; all SINEs and Alu), long interspersed nuclear elements (LINEs; L1), and long terminal repeats (LTRs; all LTRs and endogenous retrovirus 1 [ERV1]) than msHSBs and other genomic regions (Fig. 5 and *SI Appendix*, Figs. S13 and S14 and Tables S12 and S13). While the median number of bases for segmental duplications was the same for all groups (0 bp/100 kbp) (Fig. 5*A*), the underlying distributions were significantly different, as also supported by the average number of bases within segmental duplications in each category: 3.3 kbp for nonreuse EBRs, 2.3 kbp for reuse EBRs, 129 bp for msHSBs, and 813 bp for the other genomic regions (*SI Appendix*, Table S12). The biggest difference between EBRs (reuse and nonreuse) and msHSBs was found for Alu repeats, whose density in both reuse and nonreuse EBRs is ∼1.6× higher than in msHSBs. Significantly different densities within reuse and nonreuse EBRs were found for segmental duplications, LTRs (all LTRs, ERV1, and ERVL-mammalian apparent LTR retrotransposons [MaLR] independently), and SINEs (mammalian-wide intersterpsed repeats [MIR] only). For all these sequence features except segmental duplications, reuse EBRs had a higher density than nonreuse EBRs. The largest difference was observed for LTRs, with a median density ∼1.5× higher in reuse EBRs than nonreuse EBRs (Fig. 5*B*). msHSBs were found to have significantly higher densities of DNA retrotransposons (all DNA retrotransposons and hAT-Charlie independently), LINEs (L2), and SINEs (MIR) than reuse and nonreuse EBRs or other regions of the human genome.

### Analysis of the Distribution of TADs and Chromatin Compartments in msHSBs and EBRs.

**Fig. 6.**
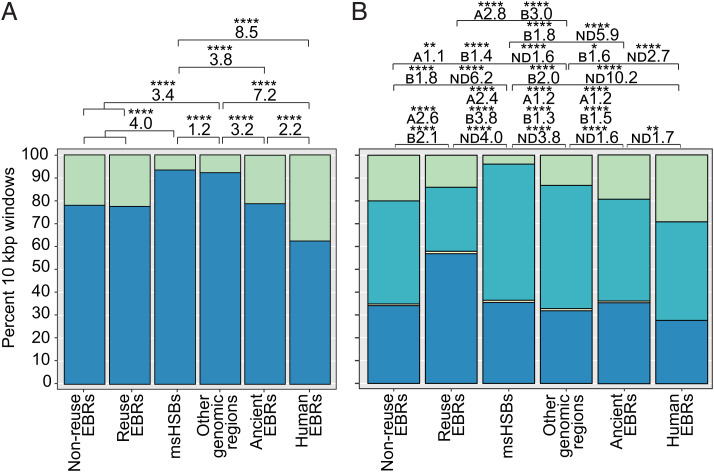
Distribution of TADs and chromatin compartments within EBRs, msHSBs, and other regions of the human genome. (*A*) Percentage of 10-kbp windows with (1+; blue) or without (0; green) defined TADs. (*B*) Percentage of 10-kbp windows in A (blue) or B (turquoise) compartments containing compartment switches (A/B; yellow) or without defined compartments (ND; green). ORs for pairwise comparisons are given above the bars. Asterisks depict Bonferroni-corrected *P* values. Only significant comparisons are shown. Total numbers of windows were 6,224 for nonreuse EBRs, 285 for reuse EBRs, 253 for human-specific EBRs (“human EBRs”; i.e., those EBRs that distinguish the human genome from the primate ancestor), 6,256 for more ancient human lineage-specific EBRs (“ancient EBRs”; EBRs identified in the branches from the mammalian ancestor until the primate ancestor), 168,649 for msHSBs, and 106,272 for the remaining human genome (“other genomic regions”). The *y*-axis scale is the same for *A* and *B*. *P < 0.05; **P ≤ 0.01; ****P ≤ 0.0001.

To investigate the relationship between msHSBs and chromatin architecture, we analyzed the distribution of TADs in 10-kbp windows within msHSBs, EBRs identified in the human lineage, and other regions of the human genome. The TADs used for these analyses were identified in human lymphoblastoid cells. We found an association between human-specific EBRs (EBRs that distinguish the human genome from the primate ancestor) and the absence of TADs, with the odds ratio (OR) of EBRs not having a TAD 8.5× higher than msHSBs (*χ*^2^ = 385.8, *P* < 2.2e-16) (Fig. 6*A*). This difference was less pronounced when we compared msHSBs with the more ancient human lineage-specific EBRs (EBRs identified in ancestors descending from the mammalian ancestor to the primate ancestor; OR = 3.8, *χ*^2^ = 1987, *P* < 2.2e-16). Human-specific EBRs had fewer TADs as compared with the more ancient human lineage-specific EBRs (OR = 2.2, *χ*^2^ = 36.2, *P* = 1.7e-09). The difference in TAD distribution within reuse and nonreuse EBRs was not significant.

We also assessed the distribution of A and B chromatin compartments within msHSBs and EBRs (Fig. 6*B*). The largest difference in A compartment colocalization was observed between reuse and nonreuse EBRs, where reuse EBRs had 2.6× higher odds of locating within an A compartment than nonreuse EBRs (*χ*^2^ = 61, *P* = 5.6e-15). Reuse EBRs also colocalized with an A compartment more often than msHSBs (OR = 2.4, *χ*^2^ = 55.8, *P* = 7.9e-14). Contrariwise, msHSBs had the highest fraction of windows colocating with a B compartment, while reuse EBRs had the lowest. The differences between the distribution of A/B transition windows (i.e., 10-kb windows having both A and B compartments) between the genomic groups were not statistically significant. There was a tendency of msHSBs to have fewer windows (4%) without a defined compartment than EBRs (≥14%) (Fig. 6*B*). The largest difference was found between human-specific EBRs and msHSBs (OR = 10.2, *χ*^2^ = 422, *P* < 2.2e-16) followed by nonreuse EBRs and msHSBs (OR = 6.2, *χ*^2^ = 3,647, *P* < 2.2e-16). All other pairwise comparisons between groups were statistically significant, except for reuse EBRs compared with other regions of the human genome and reuse compared with nonreuse EBRs (Fig. 6*B*).

## Discussion

We created a detailed, sequence-based computational reconstruction of the karyotype and syntenic relationships of the common ancestor of all mammals using representative species from all eutherian orders, three of seven marsupial orders, and the monotremes (Fig. 1). Broad sampling across the mammalian phylogeny also allowed us to reconstruct ancestral genomes at 15 other ancestral nodes of mammalian evolution, 6 for the first time to our knowledge (Euarchonta, Primatomorpha, Atlantogenata, Laurasiatheria, Scrotifera, Cetruminantia). The reconstructed ancestral mammal genome revealed that extant mammal genomes are a mosaic derived from the evolutionary shuffling of 2,557 syntenic segments, on average 880 kbp, representing 69 to 94% of the genome size of all species analyzed. These syntenic segments thus serve as the fundamental building blocks of all mammalian genomes—genomic elements analogous to those of the periodic table of chemical elements—each with conserved syntenic relationships and biological functions. Our results shed light on the functions of these genomic segments and the evolutionary mechanisms that conserve syntenic relationships over time.

We propose prezygotic purifying selection as the evolutionary mechanism preventing meiotic chromosome rearrangements that disrupt msHSBs, leading to the conservation of mammalian chromosomes observed over the entire ∼180 My of their evolutionary history. Conservation of some msHSBs was found to extend to the amniote ancestor (∼320 Mya). The significant associations of msHSBs with developmental functions (*SI Appendix*, Fig. S12) and the presence of TADs (Fig. 6*A*) strongly support this hypothesis. This postulated mechanism follows the historical view that most chromosome rearrangements are underdominant or lethal (43). Considering that human males produce 10^12^ to 10^13^ gametes in a lifetime (44), the number of potential rearrangements that could occur during meiosis on an evolutionary timescale within a species is extremely large. With stable rearrangements being relatively rare in mammalian evolution (two per million years), those that persist would likely either be neutral or impart selective advantage (45) while also becoming a reproductive isolating mechanism during speciation. That some rearrangements can be adaptive and positively selected is now clear from yeast experimental systems (46, 47) and cancer cells (14).

The reconstructed mammalian ancestor karyotype recovered >80% of mammalian BUSCOs, a curated set of single-copy orthologs present in most if not all mammalian species (*SI Appendix*, Fig. S1). This result should be close to the theoretical maximum because of the bias toward eutherian species in the BUSCO dataset and the recovery of most BUSCOs present in the human and platypus genomes in our reconstructions (*SI Appendix*, Table S5). The remaining 20% of mammalian BUSCOs could represent genes gained after the divergence of monotremes and therian mammals, mistakenly inferred to be present in all mammals. This interpretation is substantiated by the successive increase in BUSCO completeness for younger reconstructed ancestors. Comparisons between our reconstructions and those reported previously (3, 7, 9, 20, 41, 48–50) showed good agreement in chromosome numbers (±1 chromosome); however, there were differences in SF order and orientation for the eutherian and more recent ancestors (boreoeutherian, cetartiodactyl, and ruminant) and more striking differences for the mammalian and therian ancestors (*SI Appendix*, *SI Text* and Figs. S15–S20). These discrepancies are likely due to the increased taxon sampling and higher SF resolution in our study, which improved continuity of the mammalian and therian ancestor reconstructions and provided additional support for ancestral syntenies not recovered previously. Comparisons of the reconstructions based on the three reference genomes showed that they are robust, and reference bias is minimal. However, for the older (mammalian and therian) ancestors, some uncertainties remain. Deeper taxon sampling and even higher continuity assemblies may be necessary to resolve these discrepancies.

The rates of interchromosomal and intrachromosomal rearrangements differed dramatically among MAMs, with the largest ancestral MAMs more subject to fissions than the smaller MAMs (Fig. 3 and *SI Appendix*, Fig. S10). Unexpectedly, we found that four MAMs (MAM7, MAM14, MAM16, and MAM18) were maintained as a single chromosome for >150 My of mammalian evolution (until the primate and ruminant ancestors in addition to some extant species not belonging to the primate and ruminant lineages) and that these chromosomes had very small fractions of their sequence subjected to internal rearrangements (Fig. 3 and *SI Appendix*, Figs. S6 and S7). By comparing MAMs with reconstructed avian and amniote ancestor chromosomes, we were surprised to find that the reconstructed MAM karyotype shared nine complete chromosomes with the avian and amniote reconstructed ancestral karyotypes (18, 42), encompassing at least ∼320 My of vertebrate evolution (Table 1). While the evolutionary stability of microchromosomes in the vertebrate lineage was recently recognized (51), the fate of ancestral vertebrate microchromosomes in the early evolution of mammals had not been resolved until now. The higher density of protein-coding genes on the smaller mammalian ancestral chromosomes (*SI Appendix*, Table S9) suggests they would have a lower tolerance to chromosomal rearrangements due to an increased probability of disrupting genes and regulatory pathways (18, 52). Increasing evidence of the importance of three-dimensional (3D) genome organization in the nucleus and the high frequency of interchromosomal regulatory interactions (53, 54) point to another level upon which natural selection can act to maintain these ancient chromosomes as single entities.

The role of MAMX in sex determination in early mammals is unclear. We found that the eutherian X chromosome is derived from the fusion of MAMX/THEX and part of an autosome (MAM1/THE1) in the eutherian ancestor (Fig. 2), confirming previous observations (48, 55). In platypus and chicken, MAMX is orthologous to OAN6 and GGA4, respectively. Assuming that a genetic mechanism of sex determination existed in the ancestor of all mammals, our results support either MAMX or MAM2 as the ancestral mammal sex chromosome. Because MAMX was reconstructed as an independent chromosome and plays a role in sex determination in therian mammals, it may have had this function in the mammalian ancestor, and the complex sex determination system observed in monotremes evolved independently after their divergence. Alternatively, MAM2 is orthologous to sex chromosomes in the platypus (X2, X3, and X5) and the chicken (Z), which could support its sex chromosome status in the mammalian ancestor. Nonetheless, the sex chromosome organization and sex determination genes in platypus (*AMH*) and chicken (*DMRT1*) are different, which suggests independent evolution of their sex determination systems (55) and lessens the support for MAM2 as the ancestral mammalian sex chromosome.

The increase in chromosome rearrangement rate during the evolutionary period from the cetartiodactyl to ruminant ancestors following the bolide impact at the Cretaceous–Paleogene boundary followed previous reports (5, 20) (Fig. 1 and *SI Appendix*, Table S6). However, rearrangement rates in our study were lower than in Farré et al. (20) and higher than in Murphy et al. (5) (*SI Appendix*, Table S14). Compared with previous estimates in the primate lineage (3), the rates estimated herein are slightly lower between key ancestral nodes and higher for the tips of the phylogeny (*SI Appendix*, Table S14). This variability likely results from differences in assembly quality, taxon sampling, and the SF resolution of our analysis compared with previous studies. While there is high confidence in the rearrangement rates estimated for the most recent ∼100 My of mammalian evolution, from the eutherian ancestor to extant species, we urge caution in interpreting results for the early branches of mammalian evolution because of the uncertainty associated with some SF adjacencies in the mammalian and therian reconstructions.

Variation in rearrangement rates could relate to differences in genomic architecture, the type and distribution of repetitive elements, and changes in the environment known to cause chromosome rearrangements (15). Our observations that EBRs are enriched for segmental duplications and transposable elements (TEs; Fig. 5 and *SI Appendix*, Figs. S13 and S14) support the hypothesis that repetitive sequences can provide additional templates for nonallelic homologous recombination (NAHR), increasing the chances for chromosome rearrangements to occur. Further, we found that reuse EBRs have the highest densities of LINE L1, LTR ERV1, and LTR ERVL-MaLR. These TE subclasses are still active in several mammalian lineages (56, 57), including rodents, carnivores, and bovids, for which we detected the most breakpoint reuse. These observations suggest that LINEs and LTRs played an essential role in the genesis of mammalian chromosome rearrangements, particularly the repeated involvement of genomic regions in independent rearrangement events in different lineages. This hypothesis is supported by the mediator role of LINEs and LTRs in disease-related somatic rearrangements, including chromosomal translocations and deletions (58, 59). We also found that regions of the human genome harboring reuse breakpoints are more frequently in type A compartments (Fig. 6*B*), which are usually associated with transcriptionally active genes and open chromatin, thus supporting the role of open chromatin in facilitating chromosome rearrangements (24). In rodents, EBRs were found associated with postmeiotic double-strand breaks in A compartments (31). Together with observations in *Drosophila*, where somatic TE insertions are enriched in gene-dense and open chromatin regions (60), these data suggest that breakpoint reuse could result from a higher frequency of templates for NAHR caused by the frequent insertion of TEs in open chromatin regions.

Trends in the rates of chromosome rearrangements support the association between chromosome rearrangements and speciation (11). The high rate of rearrangements observed from the mammalian to the therian ancestor (Fig. 1) coincides with the oldest mammaliaform adaptive radiation that occurred in the Early to Mid-Jurassic, followed by slower rearrangement and morphological evolution rates until the end of the Cretaceous period (61, 62). The subsequent increase in rearrangement rates observed in the cattle lineage coincides with the Cretaceous–Paleogene boundary, the Paleocene–Eocene thermal maximum (63), and the extensive spread of grassland ecosystems (64) and appearance of the first ruminants ∼50 Mya (65).

A substantial fraction of the HSBs (∼70%) was conserved in all the mammalian species studied (msHSBs). These msHSBs cover at least 50% of the genome size of each species used in our analysis. msHSBs were found to contain a significantly higher fraction of genes involved in anatomical morphogenesis and the development of the central nervous system than non-HSB regions (*SI Appendix*, Fig. S12*A*), confirming our earlier findings (15). We extend these observations by showing that the msHSBs significantly overlap with TADs, as defined in the human genome (Fig. 6*A*). These results indicate that msHSBs are core functional units of chromatin structure and organization, and play a role in coordinated transcriptional control of their internal genes (66). EBRs have significantly more and smaller complete genes than msHSBs (Fig. 4*A*). EBRs are also more frequently located in regions not encompassed by TADs (Fig. 6*A*), as observed previously (15, 33, 67). The odds of EBRs not being encompassed by a TAD as defined on the human genome were substantially greater than for other regions of the human genome, but the more recent human-specific EBRs were less likely to overlap with TADs than the more ancient human lineage-specific EBRs. The difference between human lineage-specific and human-specific EBRs was likely due to the difference in the resolution of the two types of EBRs caused by alignment bias and not evolutionary constraint; the median length for human-specific EBRs is ∼63 kbp, while it is 110 kbp for nonhuman-specific EBRs in the lineage leading to humans. Nonetheless, our data support that the loss of TADs results from genome rearrangements and/or rearrangements tend to occur between TADs, as observed in gibbon (33). These findings have important implications, especially in light of a recent study showing that the chromosome-scale chromatin structure was evolutionarily conserved for over 50 My within the carnivore family (68), even after chromosome rearrangements. In addition, recent evidence points to evolutionary constraints in maintaining 3D genome structure in bilaterians (69). Our results demonstrate that maintaining the TAD structure of HSBs is important in genome evolution. By comparison, EBRs appear to function as regions of evolutionary plasticity, where novel genes, segmental duplications, and transposable elements accumulate due to NAHR, nonhomologous end joining, and other mechanisms, such as those that are activated to repair DNA at breakpoints (70).

By drawing on high-contiguity genomes from multiple genome sequencing efforts, we reconstructed 16 ancestral karyotypes along the mammalian phylogeny and performed a comprehensive study of mammalian chromosome evolution. Many ancestral syntenies were identified for the first time to our knowledge. The assignment of EBRs to different branches in the mammalian phylogeny can be useful for subsequent analyses of the relationship between genome rearrangements, gain and loss of functional elements (both coding and noncoding), effects on 3D chromosome structure during evolution, and the genomic origins of adaptive traits. With improved tools for sequencing, assembling, aligning, comparing, and visualizing large numbers of reference-quality genomes, it will soon be possible to extend sequence-based genome reconstructions deeper into evolutionary time (71) and to explore the nature and consequences of chromosome rearrangements that occurred during more recent radiations of mammals and other eukaryotes. Conservation and de-extinction efforts may also benefit from knowing ancestral karyotypes and the chromosomal orientation of conserved syntenic regions.

## Materials and Methods

### Reconstruction of Ancestral Chromosomes and Assessment of Completeness.

The complete list of genomes used in this work and their genome assembly statistics are presented in Dataset S1. Details for the newly generated or upgraded genome assemblies of the narwhal, koala, tree pangolin, rock hyrax, three-banded armadillo, and large tree shrew are described in *SI Appendix*. Divergence times and topologies were obtained from TimeTree (72). We used the human, cattle, and sloth genomes as references to produce RACFs. Scaffolds shorter than 10 kbp were removed from each target genome before alignment. For the reference genomes, only sequences assigned to chromosomes were used for alignment (details on genome alignments are in *SI Appendix*). We used DESCHRAMBLER (3) at an SF resolution of 300 kbp to reconstruct the ancestral RACFs at 16 nodes of the mammalian phylogeny. The RACFs were then assigned and ordered within reconstructed chromosomes following previously described methodologies (3, 18) (*SI Appendix* has a brief description). We evaluated genome completeness of the reconstructed RACFs using the BUSCO (version 5.2.2) software with the mammalian OrthoDB version 10 dataset (38). For mammalian ancestor RACFs, we also evaluated the presence of mammalian BUSCOs found in both the human and platypus genomes.

### Assessment of Consistency between Ancestral Reconstructions Based on Distinct Reference Genomes and Previous Reconstructions.

We identified each human SF adjacency present in the human genome-based reconstructions of the mammalian, therian, eutherian, and boreoeutherian ancestors. Next, we examined whether these adjacencies were also present in the cattle and sloth genome-based reconstructions. Each SF adjacency was classified as maintained, extra, or inconsistent as described in *SI Appendix*, and the percentage of inconsistent adjacencies was calculated (*SI Appendix*, Tables S2 and S3). For human–cattle and human–sloth overlapping reconstructed segments, we identified all inconsistent HSBs (*SI Appendix* has details). The cumulative length of these inconsistent HSBs was then divided by the total length of the reconstruction to obtain the fraction of the ancestral genome comprising inconsistencies. Both evaluations were performed at 300-kbp SF resolution.

To evaluate the recovery of previously reported ancestral syntenies, we compared the ancestral syntenies of HSAs identified on the reconstructed chromosomes with those compiled from the literature for the mammalian (7, 48), therian (7, 9, 48), eutherian (3, 7), boreoeutherian (3, 49), xenarthran (41), cetartiodactyl (20), and ruminant (bovids) (20, 50) ancestors. Comparative visualizations were generated using the syntenyPlotteR (73) R package.

### Identification of msHSBs, EBRs, and Chromosome Rearrangements.

We defined msHSBs as regions of the HSAs that completely overlap with SFs of other species. These were identified pairwise for all species with a chromosome-scale scaffold or chromosome-level genome assembly included in our dataset. Eight scaffold-based genome assemblies were not used to define msHSBs to avoid fragmentation due to their lower contiguity. Human coordinates of msHSBs were then translated to each of the other 23 mammalian genome coordinates using the Univerisy of California Santa Cruz (UCSC) liftover tool (74). The expected maximum msHSB size was calculated assuming an exponential distribution, and the distribution was compared with the observed values for msHSBs that exceeded the maximum expected size as described (15, 25).

To avoid errors associated with the manual joining of RACFs, we classified EBRs only within RACFs. We detected EBRs relative to the mammalian ancestor in all other ancestors’ RACFs and each reference genome using a published methodology (3). The number and types of chromosome rearrangements (i.e., inversions, fusions, and fissions) for each phylogenetic branch were identified by comparing each ancestral karyotype with those of more recent ancestors. This was performed using the genome rearrangements in man and mouse (GRIMM; 75) software, followed by manual curation of the results. The process was repeated for the lineages leading to human, cattle, and sloth. Breakpoint and rearrangement rates for each branch leading to human, cattle, and sloth were calculated by dividing the number of detected EBRs and rearrangements by the length of the branch in millions of years. Differences in rates compared with the average of all branches were analyzed with a Student’s *t* test as described (3). FDR-corrected *P* values were calculated using the p.adjust function from the R package (76). Reuse EBRs were identified by manually inspecting genome alignments in the UCSC genome browser (74) and Evolution Highway Comparative Chromosome Browser (77).

### Fraction of Rearranged MAMs.

We calculated the fraction of each MAM involved in intrachromosomal rearrangements as compared with other ancestors and extant descendant and out-group species as described (3, 18) (a brief explanation is in *SI Appendix*). Results were visualized using the ggplot2 (78) R package.

### Analysis of Gene Functions in EBRs and msHSBs.

Coordinates of all human protein-coding genes were downloaded from Ensembl (release 104) (79). We focused on human protein-coding genes because the subsequent analyses used human genome annotation. We assigned the genes to EBRs or msHSBs based on their coordinates in the human genome. To identify GO terms overrepresented in msHSBs, we considered msHSBs that were >1 Mbp. To evaluate functional enrichments in EBRs, we considered only those genes within EBR boundaries. We used ShinyGO (version 0.741) (80) to detect overrepresented GO terms in our datasets. We considered terms significantly enriched when FDR was <5% in EBRs or msHSBs relative to all other regions in the human genome.

### Analysis of Sequence Features in MAMs, EBRs, and msHSBs.

TADs defined in the human genome (hg38) using the GM12878 dataset (81) were downloaded from the 3D genome browser (82). A and B compartment definitions for GM12878 were obtained from Rao et al. (81) and translated from human genome version hg19 to version hg38 coordinates using the UCSC liftover tool (74). Human segmental duplication and repeat annotations were downloaded from the UCSC genome browser (74). Human protein-coding genes and human 1:1 orthologs with the cattle (bosTau9), greater horseshoe bat (mRhiFer1_v1.p), African elephant (loxAfr3), Hoffmann's two-fingered sloth (choHof1), and chicken (galGal6a) genomes were downloaded from Ensembl (release 104) (79). We used Hoffmann’s two-fingered sloth for this analysis because the gene annotation for the Southern two-toed sloth was not available. These two species belong to the same genus (*Choloepus*) and diverged only ∼8 Mya, so we assumed that the genes identified in Hoffmann’s two-fingered sloth are also present in the Southern two-toed sloth. Mammalian msHSBs were defined as described above. Only EBRs identified in the human lineage were used for subsequent analyses.

We tested for differences in human protein-coding gene density for each MAM (number of genes per MAM length) compared with the average density across all MAMs with a Student’s *t*-test statistic. *P* values were corrected for FDR using the p.adjust function from the R package (76). We tested for differences in the median gene length within human lineage-specific EBRs, msHSBs, or other regions of the human genome using the pairwise Wilcoxon rank-sum test from the base R package (76). The analysis was performed independently using the length of human genes and the length of orthologs in cattle, greater horseshoe bat, African elephant, Hoffmann's two-fingered sloth, and chicken.

For subsequent analyses of sequence features in msHSBs and EBRs, the human genome was divided into 10- and 100-kbp windows using BEDTools (version 2.29.0) (83). Windows with >50% of their length in sequence gaps or overlapping human centromeres were removed from the analysis. Each remaining window was classified as within an msHSB or an EBR if fully overlapping one of these regions or as within other regions of the human genome. The number of human protein-coding genes and bases within genes were counted for each 100-kbp window, which approximates the median length of EBRs. The number of TADs and bases within TADs, A/B compartments, segmental duplications, and repetitive sequences were counted for each 10-kbp window. Only repeat subclasses with $\bar{x}$ ≥ 100 bp per 10-bp window in msHSBs, EBRs, or other genomic regions were reported. Comparisons between msHSBs, human-specific EBRs, nonhuman-specific EBRs in the human lineage, reuse EBRs, nonreuse EBRs, and other genomic regions were performed as for the median gene lengths described above. The association between TAD absence and genomic region was tested using a Pearson χ^2^ test of independence from the R package (76). The same approach was taken for association tests involving A and B compartments. The OR for each comparison was calculated using the oddsratio function from the epitools R package (84).

## Supplementary Material

Supplementary File

Supplementary File

Supplementary File

Supplementary File

Supplementary File

Supplementary File

Supplementary File

Supplementary File

Supplementary File

Supplementary File

Supplementary File

Supplementary File

Supplementary File

## Data Availability

Sequencing data and genome assemblies used in this work are described in dataset S1 and *SI Appendix* and are publicly available.
