# Supplementary material for: Evolution of the ancestral mammalian karyotype and syntenic regions
Source: Proc Natl Acad Sci U S A. 2022 Sep 26;119(40):e2209139119. doi: 10.1073/pnas.2209139119 (PMC9550189; doi:10.1073/pnas.2209139119)
Supplement: Supplementary File [file pnas.2209139119.sapp.pdf]

PNAS

Supplementary Information for

Evolution of the ancestral mammalian karyotype and syntenic regions.

Joana Damas, Marco Corbo, Jaebum Kim, Jason Turner-Maier, Marta Farre, Denis M. Larkin, Oliver A. Ryder, Cynthia Steiner, Marlys L. Houck, Shaune Hall, Lily Shiue, Stephen Thomas, Thomas Swale, Mark Daly, Jonas Korlach, Marcela Uliano-Silva, Camila J. Mazzoni, Bruce Birren, Diane P. Genereux, Jeremy Johnson, Kerstin Lindblad-Toh, Elinor K. Karlsson, Martin T. Nweeia, Rebecca N. Johnson, Zoonomia Consortium, Harris A. Lewin*

* Harris A. Lewin, Department of Evolution and Ecology, College of Biological Sciences, University of California Davis, CA 95616, USA, Tel: +1 530-754-5098

Email: lewin@ucdavis.edu

This PDF file includes:

Supplementary text
Figures S1 to S20
Tables S1 to S14
Legends for Datasets S1 to S14
SI References

Other supplementary materials for this manuscript include the following:

Datasets S1 to S14

Contents

Supplementary Information Text 5

Materials and Methods 5

Comparison with previously reported ancestral karyotypes. 6

Genome sequencing and assembly of the narwhal (*Monodon monoceros*). 7

Genome sequencing and assembly of the koala (*Phascolarctos cinereus*). 8

Genome sequencing and assembly of the tree pangolin (*Phataginus tricuspis*). 9

Genome sequencing and assembly of the rock hyrax (*Procavia capensis*). 10

Genome sequencing and assembly of the three-banded armadillo (*Tolypeutes matacus*). 11

Genome sequencing and assembly of the large treeshrew (*Tupaia tana*). 12

Supplemental Figures 13

Fig. S1. Benchmarking universal single-copy orthologs (BUSCO) assessment of the reconstructed ancestors using the mammalian OrthoDB v10 (odb10) dataset. 13

Fig. S2. Comparison of mammalian ancestor reconstructions using the human, cattle, or sloth genomes as a reference. 14

Fig. S3. Comparison of therian ancestor reconstructions using the human, cattle, or sloth genomes as a reference. 15

Fig. S4. Comparison of eutherian ancestor reconstructions using the human, cattle, or sloth genomes as a reference. 16

Fig. S5. Comparison of boreoeutherian ancestor reconstructions using the human or cattle genomes as a reference. 17

Fig. S6. Evolution of mammalian ancestor chromosomes in the lineage leading to sloth. 18

Fig. S7. Evolution of mammalian ancestor chromosomes in the lineage leading to cattle. 20

Fig. S8. Visualization of the evolutionary history of reconstructed mammalian chromosomes based on the sloth lineage 21

Fig. S9. Visualization of the evolutionary history of reconstructed mammalian chromosomes based on the cattle lineage 22

Fig. S10. Conservation of mammalian ancestor chromosomes (MAMs) as single chromosomal units in extant species. 23

Fig. S11. Distribution of msHSB lengths in extant mammal genomes. 24

Fig. S12. Top 20 gene ontology (GO) terms enriched in (*A*) msHSBs and (*B*) EBRs. 25

Fig. S13. Distribution of major classes of repetitive sequences within EBRs, msHSBs, and other regions of the human genome. 26

Fig. S14. Distribution of repeat subclasses within EBRs, msHSBs, and other regions of the human genome 27

Fig. S15. Comparison of reconstructed mammalian ancestor chromosomes. 29

Fig. S16. Comparison of reconstructed therian ancestor chromosomes. 31

Fig. S17. Comparison of reconstructed eutherian ancestor chromosomes. 33
Fig. S18. Comparison of reconstructed boreoeutherian ancestor chromosomes. 35
Fig. S19. Comparison of reconstructed cetartiodactyl ancestor chromosomes. 36
Fig. S20. Comparison of reconstructed ruminant (bovids) ancestor chromosomes............................. 37
Supplemental Tables 38
Table S1. Statistics of the reconstructed ancestral chromosomes.. 38
Table S2. Comparison of syntenic fragment adjacencies between human and sloth genome-based ancestral reconstructions. 39
Table S3. Comparison of syntenic fragment adjacencies between human and cattle genome-based ancestral reconstructions. 40
Table S4. Statistics of ancestral chromosome regions with structural differences in reconstructions depending on the reference genome used. 41
Table S5. Recovery of complete BUSCOs common to the human and platypus genomes (*n*=6,722) in the reconstructed mammalian ancestor chromosomes using the human, cattle, and sloth genomes as a reference. 42
Table S6. Number of evolutionary breakpoint regions (EBRs), breakpoint rates (breakpoints/My) and chromosome rearrangements that occurred during mammalian evolution. 43
Table S7. Ancestral syntenies identified in the reconstructed ancestors at 1 Mbp resolution.............. 44
Table S8: Length distribution of evolutionary breakpoint regions (EBRs) in each of the studied lineages. 45
Table S9. Distribution of human protein-coding genes in the mammalian ancestor chromosomes (MAMs). 46
Table S10. Summary statistics for the identified mammalian multispecies homologous synteny blocks (msHSBs) based of their coordinates in the human genome.. 47
Table S11: Median [interquartile range] length of genes (kbp) within msHSBs, EBRs, and other regions of the human genome. 48
Table S12: Statistics for major classes of repeats within msHSBs, reuse and non-reuse EBRs, and other regions of the human genome. 49
Table S13: Statistics for subclasses of repeats within msHSBs, reuse and non-reuse EBRs, and other regions of the human genome. 50
Table S14: Comparison of breakpoint rate estimates (breakpoints/My) to previous reports................ 51
Legends for Dataset S1 to S14 52
Dataset S1 (separate file). Statistics for the genome assemblies of descendant and outgroup species. 52
Dataset S2 (separate file). Genome alignment coverage statistics... 52
Dataset S3 (separate file). Manually curated RACFs for human genome-based reconstructed ancestors. 52

Dataset S4 (separate file). Manually curated RACFs for cattle genome-based reconstructed ancestors. 52
Dataset S5 (separate file). Manually RACFs for sloth genome-based reconstructed ancestors........... 52
Dataset S6 (separate file). Syntenic fragment coverage of the reconstructed mammalian ancestor, descendant, and outgroups species' genomes. 52
Dataset S7 (separate file). Evolutionary breakpoint regions identified in the human lineage................ 52
Dataset S8 (separate file). Evolutionary breakpoint regions identified in the sloth lineage. 52
Dataset S9 (separate file). Evolutionary breakpoint regions identified in the cattle lineage. 52
Dataset S10 (separate file). Mammalian multispecies homologous synteny blocks longer than 300 Kbp as identified in the human genome. 52
Dataset S11 (separate file). Gene ontology terms enriched in mammalian multispecies homologous synteny blocks and evolutionary breakpoint regions identified in the human lineage. 52
Dataset S12 (separate file). Orthology maps for each pairwise comparison on the human lineage. 52
Dataset S13 (separate file). Orthology maps for each pairwise comparison on the sloth lineage. 52
Dataset S14 (separate file). Orthology maps for each pairwise comparison on the cattle lineage........ 52
SI References 53
Zoonomia authors list........ 54

Supplementary Information Text

Materials and Methods

Whole-genome alignment parameters. We generated whole-genome pairwise alignments for each of the mammalian and outgroup genomes to each of the references (human, cattle, and sloth) using LastZ (v1.04) (1) with the following parameters: C=0 E=30 H=2000 K=3000 L=2200 O=400. Next, we converted the pairwise alignments to the UCSC chain and net formats with axtChain (parameters: -minScore=1000 -verbose=0 -linearGap=medium for placental mammals or -linearGap=loose for marsupials, monotremes, and outgroups) followed by *chainAntiRepeat*, *chainSort*, *chainPreNet*, *chainNet* and *netSyntenic*, all with default parameters (2). Pairwise whole-genome alignment coverages are presented in Dataset S2.

Assembly of ancestral chromosomes. To reduce the fragmentation of the reconstructed mammalian ancestors' genomes, we followed similar methodologies to those used by us previously (3, 4). Briefly, for each reconstructed ancestor, we ordered RACFs by connecting those with adjacencies that were supported by a low number of outgroup and descendant species or other phylogenetically close ancestors. For the mammalian ancestor, we first merged those RACFs that had adjacencies supported (spanned) by a chromosome or scaffold of one or both outgroup species and at least one mammalian species. Segment letter notation in Datasets S3, S4, and S5 depict fragments of the same ancestral chromosome where ordering was not fully resolved.

Classification of SFs and HSBs for assessment of consistency between reconstructions based on different reference genomes. Each SF adjacency present in the human genome-based reconstruction was defined as *maintained* if it was also present in the reconstruction being compared. *Extra* was defined as when one or both of the SFs involved in the adjacency were not present in the reconstruction being compared, or they were present, and the ends involved in the adjacency were free RACF ends. *Inconsistent* was defined as SF adjacencies that were present in the reconstruction being compared, but the end involved in the adjacency was connected to the end of a different SF or the opposite end of the same SF.

HSBs were flagged as inconsistent between reconstructions if a) they were inverted in one reconstruction relative to the other, b) if their relative position on a chromosome was different, or c) if they located on a different chromosome.

Fraction of rearranged mammalian ancestor chromosomes. Chromosome orientation in the ancestral state was established by determining which orientation would require the least rearrangements. Then we calculated the fraction involved in rearrangements by dividing the non-ancestral orientation by the cumulative length of the blocks mapped to that chromosome. The fraction of the mammalian ancestor chromosomes affected by interchromosomal rearrangements was calculated by dividing the cumulative length of the blocks of each species or reconstructed chromosome that is orthologous to each mammalian ancestor chromosome by the total length of the mammalian ancestor chromosome. The represented fraction corresponds to the lowest obtained value.

Comparison with previously reported ancestral karyotypes.

The reconstructed mammalian ancestor karyotype has ten fewer chromosomes than that produced by Zhou and collaborators (*n*=30; Fig. S15)(15), who used just four mammalian and two outgroup species for their reconstruction. The therian ancestral karyotype (*n*=18) has only one less chromosome than the previous comparative gene map (9) and sequence-based reconstructions (15) but a very different arrangement of syntenic fragments (SFs) (Fig. S16). Differences between our reconstructions and those done previously can be due to the higher number of monotreme and marsupial representative species in our study, thus reducing fragmentation of the reconstructed mammalian and therian ancestral chromosomes.

The reconstructed eutherian (*n*=20) and boreoeutherian (*n*=23) karyotypes have one less chromosome than reported previously (3). This difference resulted from joining the previous reconstructions of eutherian chromosomes (EUTs) 6 and 10 into the newly reconstructed EUT2 (Fig. S17) and the previously reconstructed boreoeutherian chromosomes (BORs) 11 and 8b into the new reconstruction of BOR7 (Fig. S18). Our data support the ancestral association of human chromosomes 10-12-22 in these two ancestors, which was not detected in the earlier study, and is substantiated by the genomes of platypus, marsupials, afrotherians, cat, and the large treeshrew.

The reconstructed cetartiodactyl ancestor karyotype (n=24) has one fewer chromosome than reported previously (19), combining CET11 and 25 into the newly reconstructed CET8 (Fig. S19). This novel join is supported by the genomes of the four species in our dataset: cattle, goat, narwhal, and pig. The chromosome number of the reconstructed ruminant ancestor karyotype (n=30) is identical to that reported previously (19) (Fig. S20); however, the order and orientation of many syntenic segments differ.

Genome sequencing and assembly of the narwhal (*Monodon monoceros*).

Species taxonomy. Eukaryota; Metazoa; Chordata; Craniata; Vertebrata; Euteleostomi; Tetrapoda; Amniota; Mammalia; Theria; Eutheria; Boreoeutheria; Laurasiatheria; Artiodactyla; Cetacea; Odontoceti; Monodontidae; Monodon; *Monodon monoceros* Linnaeus, 1758 (NCBI:txid40151).

Genome sequence report. The genome of the narwhal was sequenced from a male specimen collected from the Milne Inlet, Baffin Island, Qikiqtaaluk Region of Nunavut, Canada. A total of 253 Gb of Pacific Biosciences single molecule long reads were generated. Primary assembly contigs were obtained with FALCON-Unzip, cleaned from duplicated haplotypes using purge_dups, and phased with FALCON-Phase, using previously generated Hi-C data. The primary FALCON-Phase genome assembly was then scaffolded in HiRise (v.2.1) using Dovetail Genomics Omni-C data. The final assembly has a total length of 2.3 Gb in 100 sequence scaffolds with a scaffold N50 of 108 Mb. The majority, 99%, of the assembly sequence was assigned to 22 chromosomal-level scaffolds likely representing the 21 autosomes and X chromosome of the narwhal. The assembly has a BUSCO (v.5.0.0; (5)) completeness of 95% using the mammalia_odb10 reference set. While not fully phased, the assembly deposited is of one haplotype.

Data availability for the narwhal genome assembly.

Project accession data	
**BioProject**	PRJNA520934
**BioSample ID**	SAMN10872456
Raw data accessions	
**Pacific Biosciences CLR**	PRJNA520934
**Dovetail Genomics Omni-C**	PRJNA520934
Genome assembly	
**Assembly ID**	NGI_Narwhal_2
**Assembly accession**	SIHG02000000
**BUSCO genome score**	C:95.0% [S:93.0%, D:2.0%], F:1.5%, M:3.5%, n:9,226

Genome sequencing and assembly of the koala (*Phascolarctos cinereus*).

Species taxonomy. Eukaryota; Metazoa; Chordata; Craniata; Vertebrata; Euteleostomi; Tetrapoda; Amniota; Mammalia; Theria; Metatheria; Diprotodontia; Phascolarctidae; Phascolarctos; *Phascolarctos cinereus* (NCBI:txid184229).

Genome sequence report. We improved the contiguity of the phaCin_unsw_v4.1 koala genome assembly by scaffolding it with Dovetail Genomics Hi-C data using HiRise (v.2.1). An estimated 16,853-fold physical coverage of Hi-C data was used. The upgraded koala genome assembly has a total length of 3.2 Gb in 1,246 sequence scaffolds with a scaffold N50 of 428 Mb. The majority, 99%, of the assembly sequence was assigned to 9 chromosomal-level scaffolds likely representing the 7 autosomes and X chromosome of the koala. The assembly has a BUSCO (v.5.0.0; (5)) completeness of 94% using the mammalia_odb10 reference set. The assembly deposited is of a diploid genome.

Data availability for the tree pangolin genome assembly.

Project accession data	
**BioProject**	PRJNA359763
**BioSample ID**	SAMN06198159
Raw data accessions	
**Dovetail Genomics Hi-C**	SRR13196480
Genome assembly	
**Assembly ID**	phaCin_HiC
**Assembly accession**	POVN02000000
**BUSCO genome score**	C:94.0% [S:92.4%, D:1.6%], F:1.3%, M:4.7%, n:9,226

Genome sequencing and assembly of the tree pangolin (*Phataginus tricuspis*).

Species taxonomy. Eukaryota; Metazoa; Chordata; Craniata; Vertebrata; Euteleostomi; Tetrapoda; Amniota; Mammalia; Theria; Eutheria; Boreoeutheria; Laurasiatheria; Pholidota; Manidae; Phataginus; *Phataginus tricuspis* Rafinesque, 1821 (NCBI:txid358128).

Genome sequence report. We improved the contiguity of the ManTri_v1_BIUU tree pangolin genome assembly by scaffolding it with Dovetail Genomics Chicago data using HiRise (v.2.1). An estimated 78-fold physical coverage of Chicago data was used. The upgraded tree pangolin genome assembly has a total length of 3.0 Gb in 1,935,248 sequence scaffolds with a scaffold N50 of 10 Mb. The assembly has a BUSCO (v.5.0.0; (5)) completeness of 94% using the mammalia_odb10 reference set. The assembly deposited is of a diploid genome.

Data availability for the tree pangolin genome assembly.

Project accession data	
**BioProject**	PRJNA399460
**BioSample ID**	SAMN07678093
**Dovetail Genomics Chicago**	SUB8714060
**Assembly ID**	mPhaTri1
**Assembly accession**	SOZM02000000
**BUSCO genome score**	C:93.9% [S:92.8%, D:1.1%], F:1.6%, M:4.5%, n:9,226

Genome sequencing and assembly of the rock hyrax (*Procavia capensis*).

Species taxonomy. Eukaryota; Metazoa; Chordata; Craniata; Vertebrata; Euteleostomi; Tetrapoda; Amniota; Mammalia; Theria; Eutheria; Afrotheria; Hyracoidea; Procaviidae; Procavia; *Procavia capensis* Pallas, 1766 (NCBI:txid1973248).

Genome sequence report. We improved the contiguity of the ProCapCap_v2_BIUU_UCD rock hyrax genome assembly by scaffolding with DNA Zoo Hi-C data using the Juicer pipeline. The upgraded rock hyrax genome assembly has a total length of 3.7 Gb in 825,181 sequence scaffolds with a scaffold N50 of 135 Mb. The majority, 90%, of the assembly sequence was assigned to 27 chromosomal-level scaffolds likely representing the 26 autosomes and X of the rock hyrax. The assembly has a BUSCO (v.5.0.0; (5)) completeness of 95% using the mammalia_odb10 reference set. The assembly deposited is of a diploid genome.

Data availability for the rock hyrax genome assembly.

Project accession data	
**BioProject**	PRJNA399414
**BioSample ID**	SAMN07678107
Raw data accessions	
**DNA Zoo Hi-C**	SRX5415919, SRX5415916
Genome assembly	
**Assembly ID**	mProCap1
**Assembly accession**	PVIO03000000
**BUSCO genome score**	C:94.8% [S:94.1%, D:0.7%], F:1.3%, M:3.9%, n:9,226

Genome sequencing and assembly of the three-banded armadillo (*Tolypeutes matacus*).

Species taxonomy. Eukaryota; Metazoa; Chordata; Craniata; Vertebrata; Euteleostomi; Tetrapoda; Amniota; Mammalia; Theria; Eutheria; Xenarthra; Cingulata; Chlamyphoridae; Tolypeutes; Tolypeutes matacus (NCBI:txid183749)

Genome sequence report. The genome of the three-banded armadillo was sequenced from a fibroblast cell-line generated from a skin biopsy of a male specimen. The sample was provided by the San Diego Zoo Institute for Conservation Research. A total of ~46-fold coverage of 10X Genomics data were generated and assembled with SuperNova (v2.1.1). The resulting assembly was further scaffolded with ~54-fold physical coverage Dovetail Genomics Chicago data with HiRise (v.2.1). The final assembly has a total length of 3.8 Gb in 54,589 sequence scaffolds with a scaffold N50 of 12 Mb. The assembly has a BUSCO (v.5.0.0; (5)) completeness of 96% using the mammalia_odb10 reference set. The assembly deposited is of a diploid genome.

Data availability for the three-banded armadillo genome assembly.

Project accession data	
**BioProject**	PRJNA781997
**BioSample ID**	SAMN07678115
Raw data accessions	
**10X Genomics**	PRJNA781997
**Dovetail Genomics Chicago**	PRJNA781997
Genome assembly	
**Assembly ID**	mTolMat1
**Assembly accession**	JAKSZT010000000
**BUSCO genome score**	C:95.6% [S:88.6%, D:7.0%], F:1.1%, M:3.3%, n:9,226

Genome sequencing and assembly of the large treeshrew (*Tupaia tana*).

Species taxonomy. Eukaryota; Metazoa; Chordata; Craniata; Vertebrata; Euteleostomi; Tetrapoda; Amniota; Mammalia; Theria; Eutheria; Boreoeutheria; Euarchontoglires; Scandentia; Tupaiidae; Tupaia; *Tupaia tana* Raffles, 1821 (NCBI:txid70687)

Genome sequence report. The genome of the large treeshrew was sequenced from a fibroblast cell-line generated from a skin biopsy of a male specimen. The sample was provided by the San Diego Zoo Institute for Conservation Research. A total of ~19-fold Pacific Biosciences HiFi single molecule long reads were generated. Primary assembly contigs were obtained with FALCON-Unzip and scaffolded with 10X Genomics, and Dovetail Genomics Hi-C data using Scaff10X and HiRise, respectively. The final assembly has a total length of 2.9 Gb in 1,197 sequence scaffolds with a scaffold N50 of 113 Mb. The majority, 91%, of the assembly sequence was assigned to 28 chromosomal-level scaffolds likely representing the 28 autosomes of the large treeshrew. The assembly has a BUSCO (v.5.0.0; (5)) completeness of 97% using the mammalia_odb10 reference set. While not fully phased, the assembly deposited is of one haplotype.

Data availability for the large treeshrew genome assembly.

Project accession data	
**BioProject**	PRJNA782001
**BioSample ID**	SAMN07678117
Raw data accessions	
**Pacific Biosciences HiFi**	PRJNA782001
**10X Genomics**	PRJNA782001
**Dovetail Genomics Hi-C**	PRJNA782001
Genome assembly	
**Assembly ID**	mTupTan1
**Assembly accession**	JAKSZU010000000
**BUSCO genome score**	C:96.5% [S:92.5%, D:4.0%], F:0.8%, M:2.7%, n:9,226

Supplemental Figures

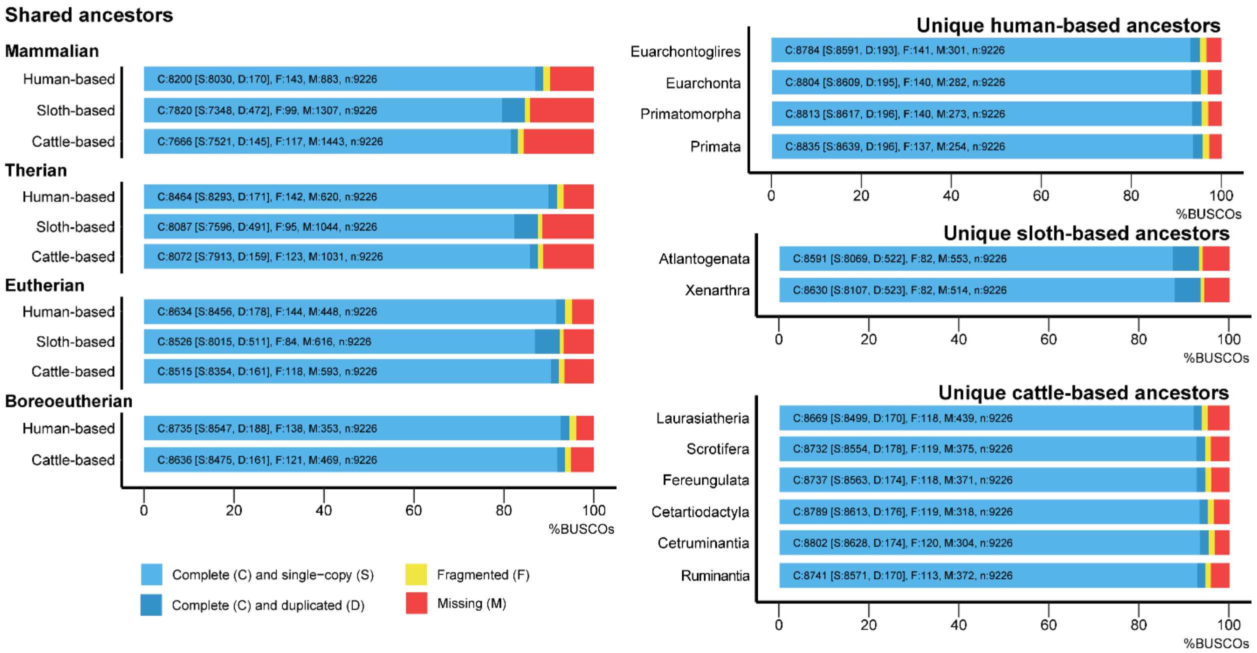

Fig. S1. Benchmarking universal single-copy orthologs (BUSCO) assessment of the reconstructed ancestors using the mammalian OrthoDB v10 (odb10) dataset.

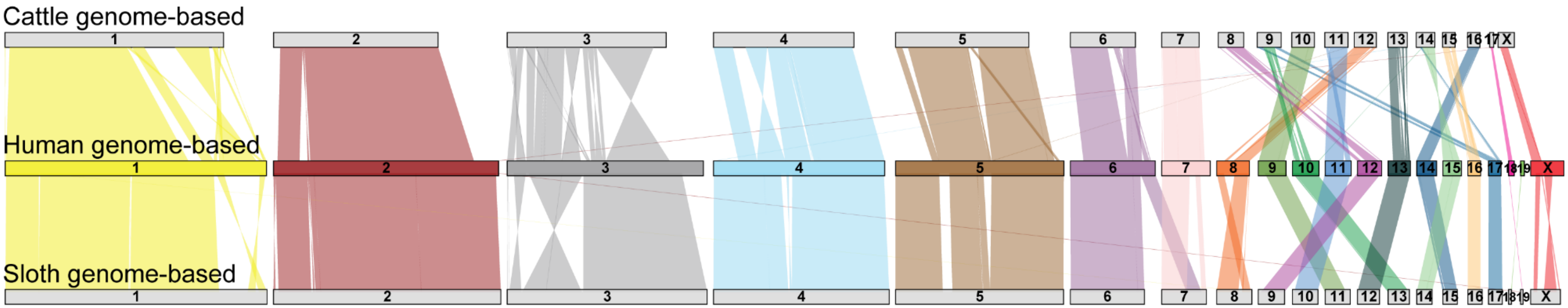

Fig. S2. Comparison of mammalian ancestor reconstructions using the human, cattle, or sloth genomes as a reference.

Human genome-based reconstructed mammalian ancestor chromosomes are distinguished by colored blocks. Grey blocks depict mammalian ancestor chromosomes reconstructed using the cattle (top) or sloth (bottom) genomes as a reference. Colored ribbons depict orthology of each human genome-based mammalian ancestor chromosome to the cattle or sloth genome-based reconstructed ancestral mammal chromosomes.

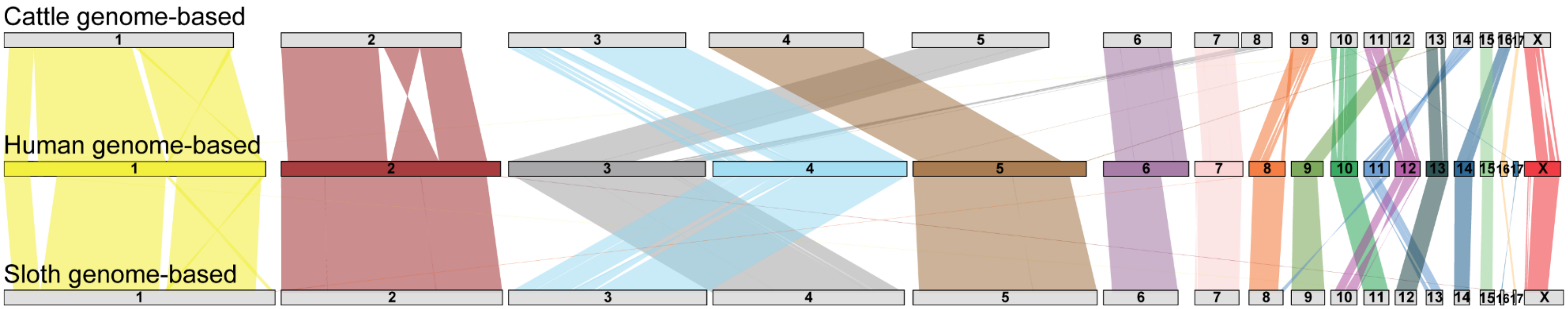

Fig. S3. Comparison of therian ancestor reconstructions using the human, cattle, or sloth genomes as a reference.

Human genome-based reconstructed therian ancestor chromosomes are distinguished by colored blocks. Grey blocks depict therian ancestor chromosomes reconstructed using the cattle (top) or sloth (bottom) genomes as a reference. Colored ribbons depict orthology of each human genome-based therian ancestral chromosome to the cattle or sloth genome-based reconstructed therian ancestral chromosomes.

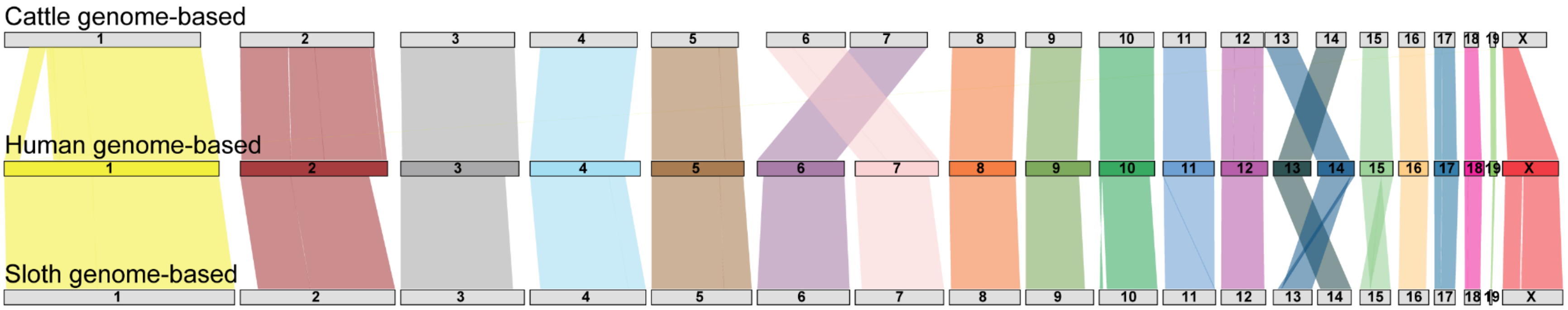

Fig. S4. Comparison of eutherian ancestor reconstructions using the human, cattle, or sloth genomes as a reference.

Human genome-based reconstructed eutherian ancestor chromosomes are distinguished by colored blocks. Grey blocks depict eutherian ancestor chromosomes reconstructed using the cattle (top) or sloth (bottom) genomes as a reference. Colored ribbons depict orthology of each human genome-based eutherian ancestor chromosome to cattle or sloth genome-based reconstructed eutherian ancestral chromosomes.

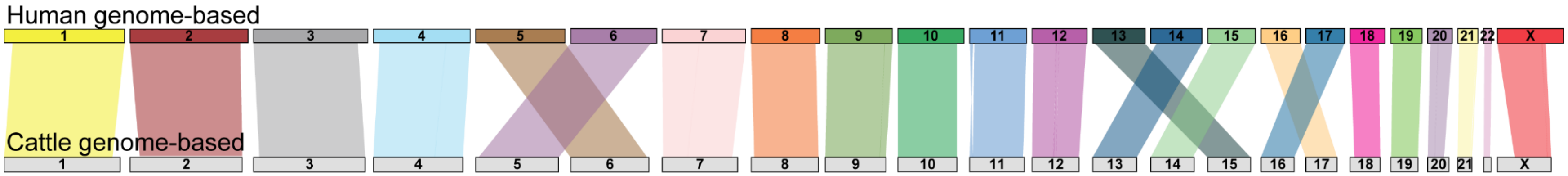

Fig. S5. Comparison of boreoeutherian ancestor reconstructions using the human or cattle genomes as a reference.

Human genome-based reconstructed boreoeutherian ancestor chromosomes are distinguished by colored blocks. Grey blocks depict boreoeutherian ancestor chromosomes reconstructed using the cattle genome as a reference. Colored ribbons depict orthology of each human genome-based boreoeutherian ancestor chromosome to the cattle genome-based reconstructed boreoeutherian ancestral chromosomes.

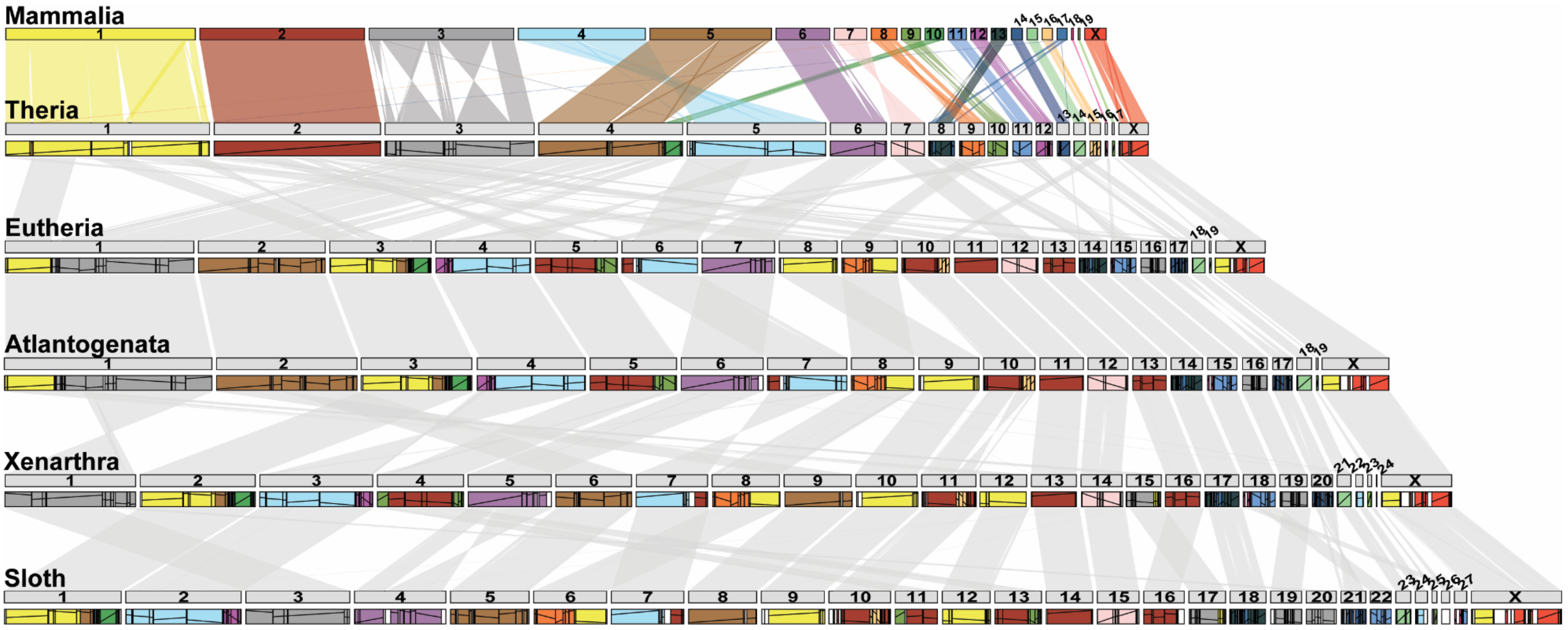

Fig. S6. Evolution of mammalian ancestor chromosomes in the lineage leading to sloth.

Mammalian ancestor chromosomes are distinguished by colors at the top of the diagram. Colored blocks for other ancestors and sloth depict orthology to mammalian ancestor chromosomes. Lines within colored blocks represent block orientation compared to the mammalian ancestor chromosomes, with positive and negative slopes portraying the same and different orientations, respectively. Grey ribbons depict orthology of each ancestor chromosome to the chromosomes of its descendant ancestor or species. An orthology map for each pairwise comparison is presented in Dataset S13.

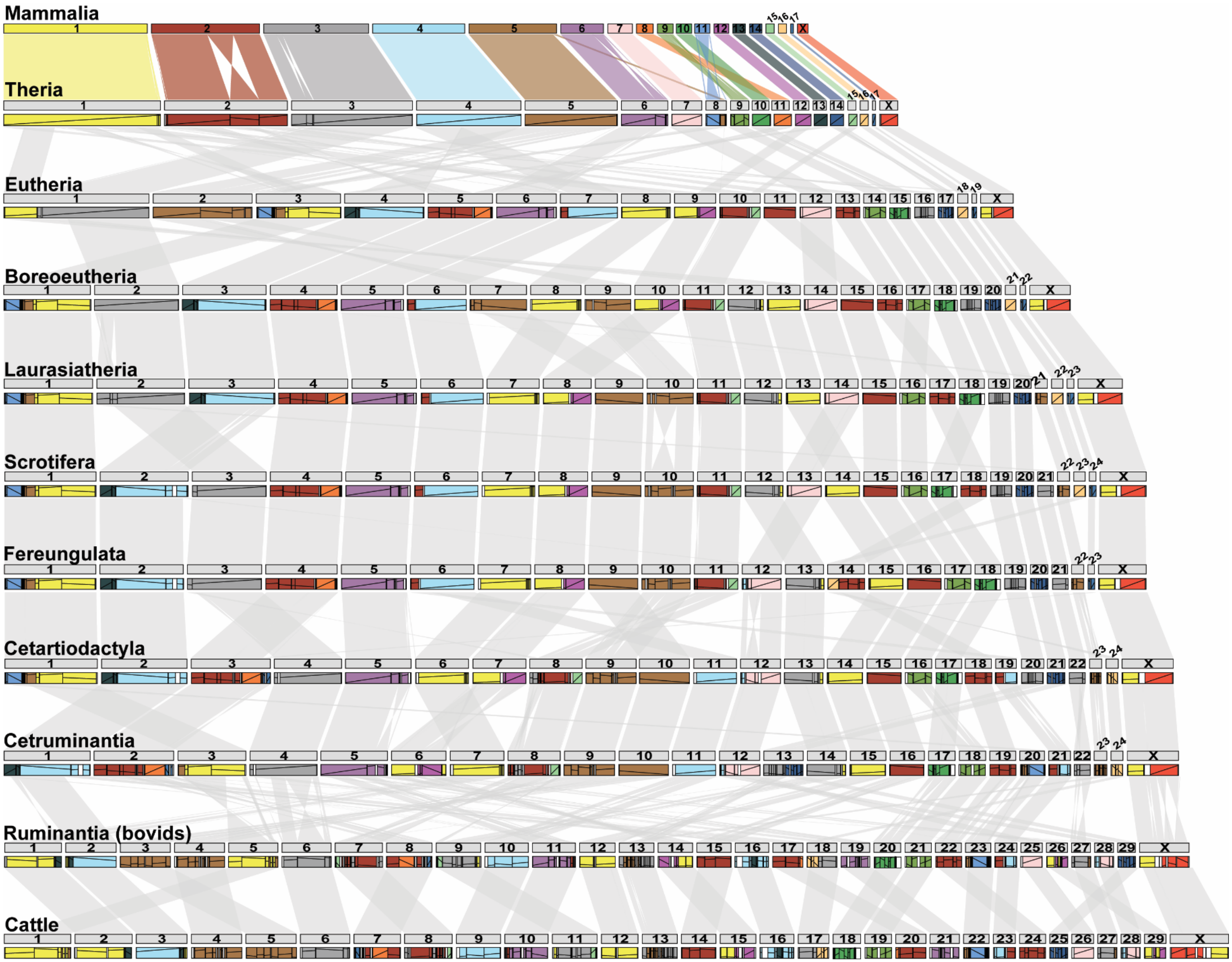
Mammalia
Theria
Eutheria
Boreoeutheria
Laurasiatheria
Scrotifera
Fereungulata
Cetartiodactyla
Cetruminantia
Ruminantia (bovids)
Cattle

Fig. S7. Evolution of mammalian ancestor chromosomes in the lineage leading to cattle.

Mammalian ancestor chromosomes are distinguished by colors at the top of the diagram. Colored blocks for other ancestors and cattle depict orthology to mammalian ancestor chromosomes. Lines within colored blocks represent block orientation compared to the mammalian ancestor chromosomes, with positive and negative slopes portraying the same and different orientations, respectively. Grey ribbons depict orthology of each ancestor chromosome to the chromosomes of its descendant ancestor or species. An orthology map for each pairwise comparison is presented in Dataset S14.

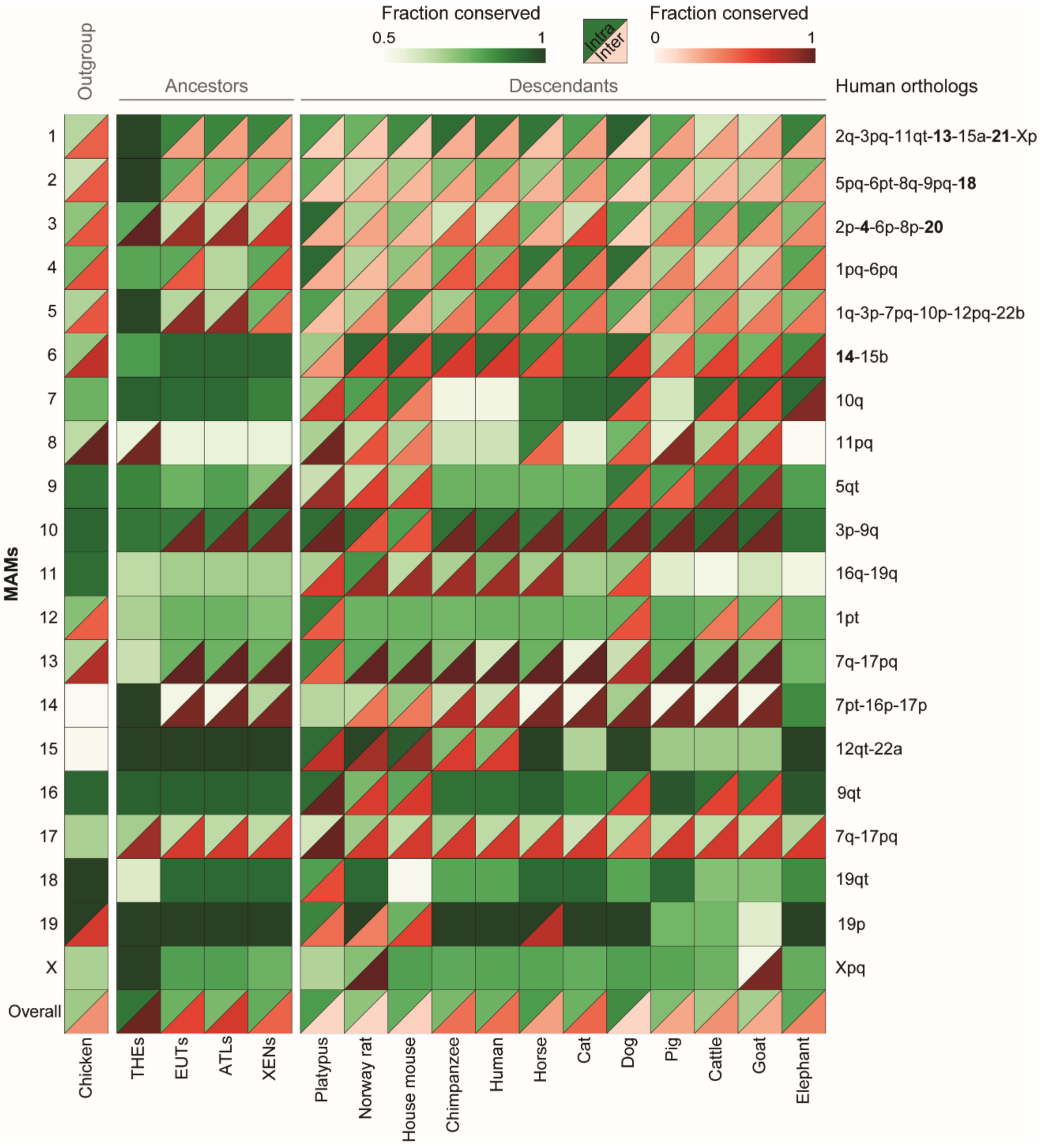

Fig. S8. Visualization of the evolutionary history of reconstructed mammalian chromosomes based on the sloth lineage.

Solid green squares indicate mammalian chromosomes that were maintained as a single synteny block (either as a single chromosome or fused with another mammalian ancestor chromosome), with shades of the color indicating the fraction of the chromosome affected by intrachromosomal rearrangements (lightest shade is most affected). Split blocks demarcate mammalian chromosomes that were affected by interchromosomal rearrangements. Upper (green) triangles show the fraction of the chromosome affected by intrachromosomal rearrangements and lower (red) triangles the fraction affected by interchromosomal rearrangements. Syntenic relationships of each mammalian ancestor chromosome to the human genome are given at the right of the diagram. MAMX appears split in goat because its X chromosome is assembled as two separate fragments. MAMs, mammalian ancestor chromosomes; THEs, therian ancestor chromosomes; EUTs, eutherian ancestor chromosomes; ATLs, Atlantogenata ancestor chromosomes; XENs, Xenarthra ancestor chromosomes.

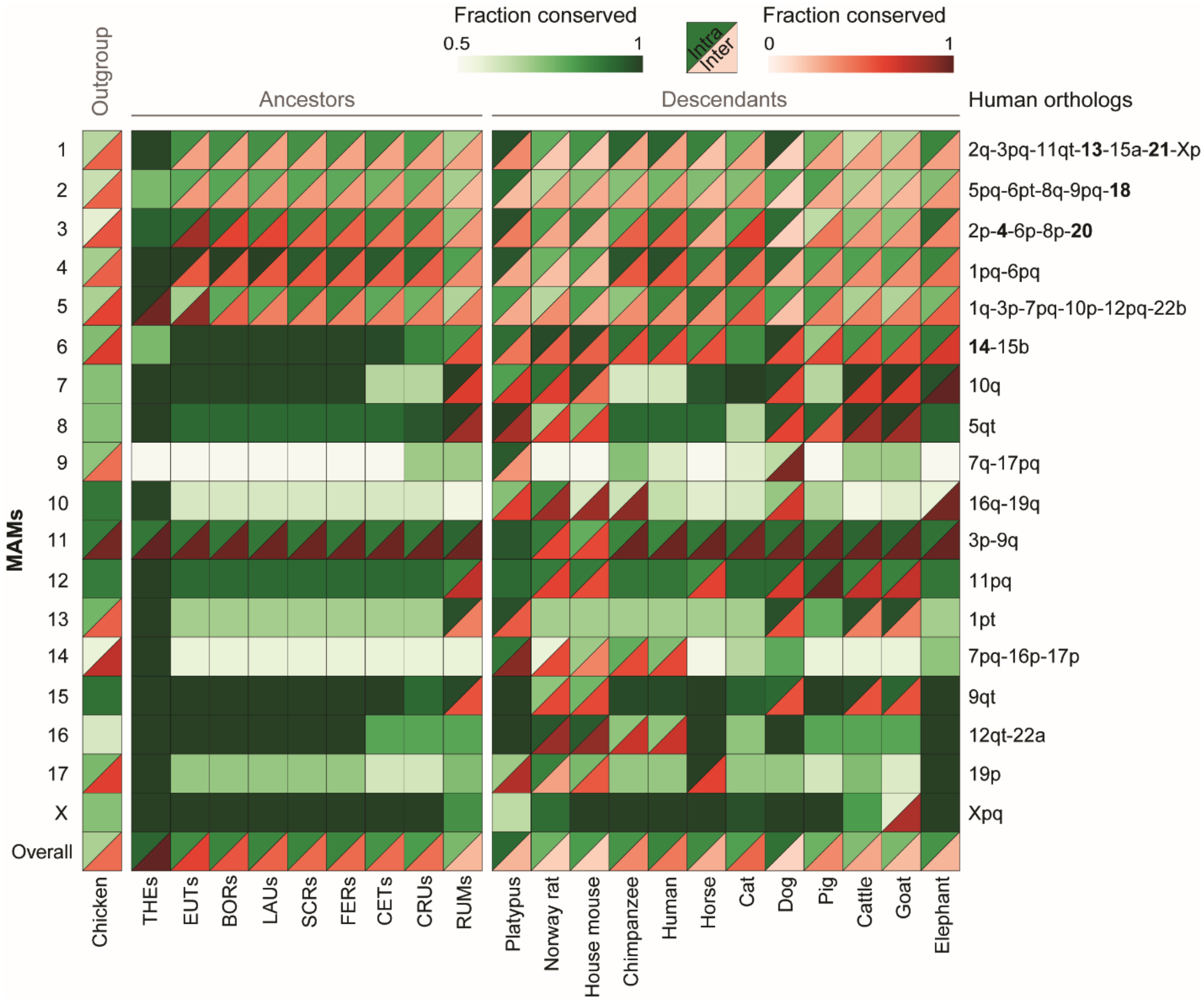

Fig. S9. Visualization of the evolutionary history of reconstructed mammalian chromosomes based on the cattle lineage.

Solid green squares indicate mammalian chromosomes that were maintained as a single synteny block (either as a single chromosome or fused with another mammalian ancestor chromosome), with shades of the color indicating the fraction of the chromosome affected by intrachromosomal rearrangements (lightest shade is most affected). Split blocks demarcate mammalian chromosomes that were affected by interchromosomal rearrangements. Upper (green) triangles show the fraction of the chromosome affected by intrachromosomal rearrangements and lower (red) triangles the fraction affected by interchromosomal rearrangements. Syntenic relationships of each mammalian ancestor chromosome to the human genome are given at the right of the diagram. MAMX appears split in goat because its X chromosome is assembled as two separate fragments. MAMs, mammalian ancestor chromosomes; THEs, therian ancestor chromosomes; EUTs, eutherian ancestor chromosomes; BORs, boreoeutherian ancestor chromosomes; LAUs, laurasiatherian ancestor chromosomes; SCRs, Scrotifera ancestor chromosomes; FERs, Fereungulata ancestor chromosomes; CETs, Cetartiodactyla ancestor chromosomes; CRUs, Cetruminantia ancestor chromosomes; RUMs, Ruminantia (bovids) ancestor chromosomes.

MAM	Chicken	Chinese alligator	Platypus	Gray short-tailed opossum	Wombat	Koala	Norway rat	House mouse	European rabbit	Large treeshrew	Chimpanzee	Human	Greater horseshoe bat	Horse	Southern white rhinoceros	Cat	Dog	Pig	Narwhal	Cattle	Goat	Southern two-toed sloth	African elephant	Manatee	Rock hyrax	Aardvark
1	4	4	11	3	4	4	12	12	7	9	8	7	12	10	10	7	14	7	7	9	9	8	10	11	11	6
2	2	2	9	2	8	1	9	9	5	7	5	5	6	7	8	5	10	8	5	7	8	6	5	5	5	4
3	5	5	16	3	4	4	9	10	4	7	5	5	7	6	7	3	12	6	6	7	7	5	9	8	9	3
4	7	7	12	2	2	2	11	10	3	3	2	2	5	7	7	4	9	7	4	5	6	3	4	3	3	1
5	2	4	9	3	6	3	12	14	8	8	6	6	8	6	6	4	9	6	6	7	7	3	5	5	5	3
6	2	3	6	1	1	2	5	5	3	5	2	2	2	2	2	1	4	2	1	2	2	1	2	3	4	1
7	1	1	2	1	1	1	5	5	1	1	1	1	1	1	1	1	3	1	1	2	2	3	2	2	1	1
8	1	2	1	1	1	1	2	3	1	2	1	1	1	2	2	1	2	2	1	2	2	1	1	1	1	1
9	1	1	4	2	2	2	2	2	1	1	2	2	1	2	2	1	4	1	1	1	1	2	1	2	2	1
10	2	3	5	1	2	1	2	2	2	2	2	2	2	2	2	2	3	2	2	2	2	2	1	2	3	2
11	1	1	3	1	1	1	5	4	2	4	2	2	4	2	2	2	2	3	2	2	2	2	1	2	2	2
12	1	1	3	2	1	2	3	4	2	2	1	1	1	1	1	1	3	2	1	3	3	3	1	1	2	1
13	2	2	2	1	1	1	1	1	1	2	1	1	1	1	1	1	3	1	1	3	3	1	1	1	1	1
14	1	1	2	1	1	1	4	4	1	1	2	2	1	1	1	1	1	1	1	1	1	1	1	1	1	1
15	1	2	3	1	1	1	3	5	2	2	3	3	3	2	2	2	2	2	2	2	2	2	1	2	2	2
16	1	1	2	1	1	1	2	2	1	2	1	1	1	1	1	1	2	1	1	2	2	1	1	1	1	1
17	1	1	1	2	2	2	2	2	3	3	2	2	2	2	2	2	3	2	2	2	2	2	2	2	3	2
18	2	2	3	1	1	1	6	4	2	2	1	1	1	2	2	1	1	1	1	1	1	1	1	1	1	1
19	1	1	2	2		1	1	1	1	1	1	1	1	1	1	1	1	1	1	1	1	1	1	1	1	1
X	1	1	1	1	1	1	2	1	1	1	1	1	1	1	1	1	1	1	1	1	2	2	1	1	1	1

Fig. S10. Conservation of mammalian ancestor chromosomes (MAMs) as single chromosomal units in extant species.

Only extant species with a chromosome or C-scaffold genome assembly are shown. The number in each cell represents the total number of chromosomal segments orthologous to each MAM in the extant genome. Dark green represents MAMs maintained as a whole chromosome or C-scaffold in the extant species' genome. Light green represents MAMs maintained as contained units, i.e., whole MAMs that have been fused to other MAMs without a break in synteny in the extant genome. Orange represents whole MAMs that have been fused to other MAMs with a break in synteny in the extant mammal genome. Red represents MAMs orthologous to multiple chromosomes or C-scaffolds in the extant genome. Grey represents MAMs without identified orthology in the extant genome.

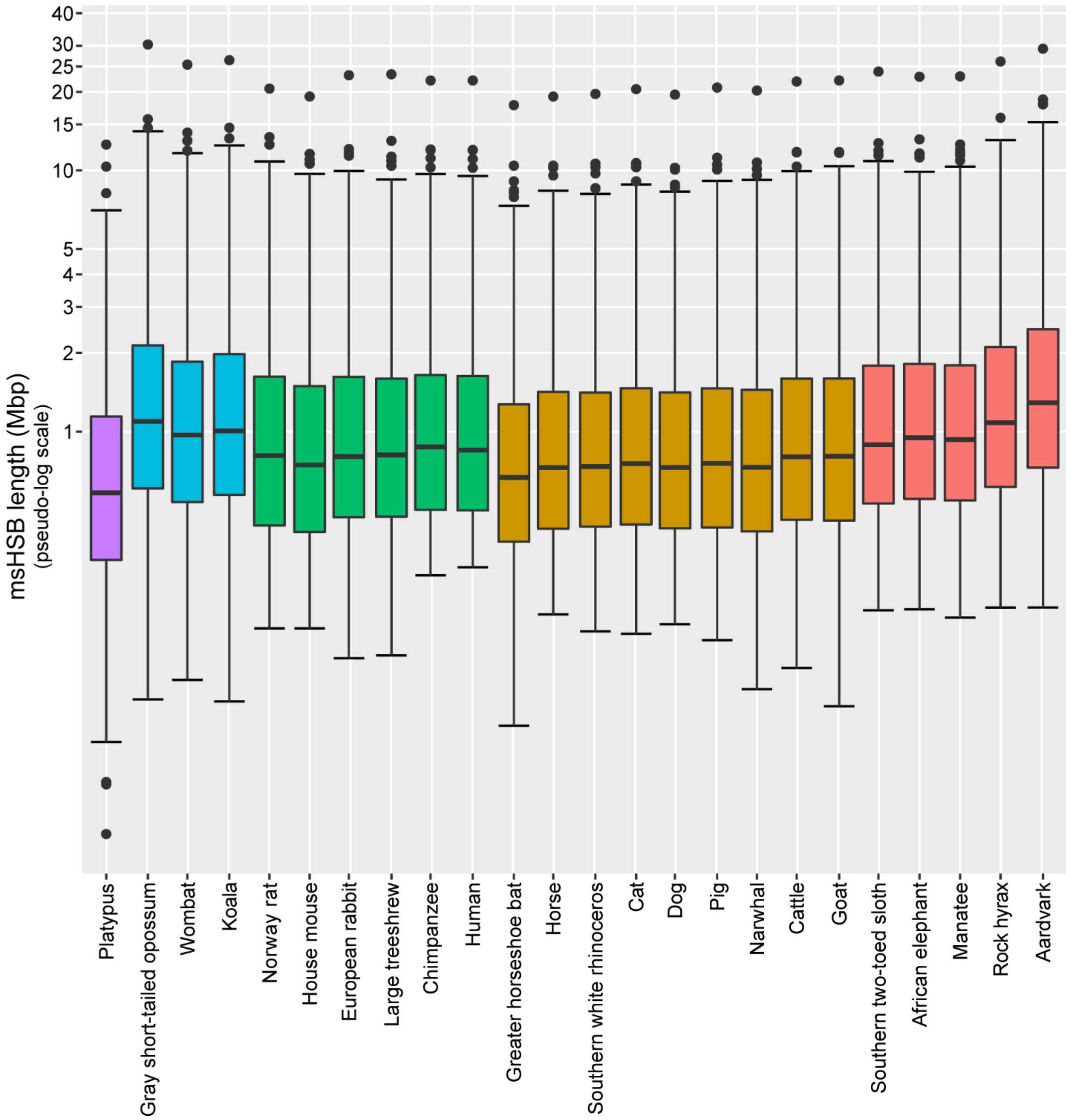

Fig. S11. Distribution of msHSB lengths in extant mammal genomes.

Only extant species with a chromosome or C-scaffold genome assembly are shown. Boxplot colors represent mammalian lineages: purple, monotremes; blue, marsupials; green, Euarchontoglires; yellow, scrotiferans, and red, atlantogenatans.

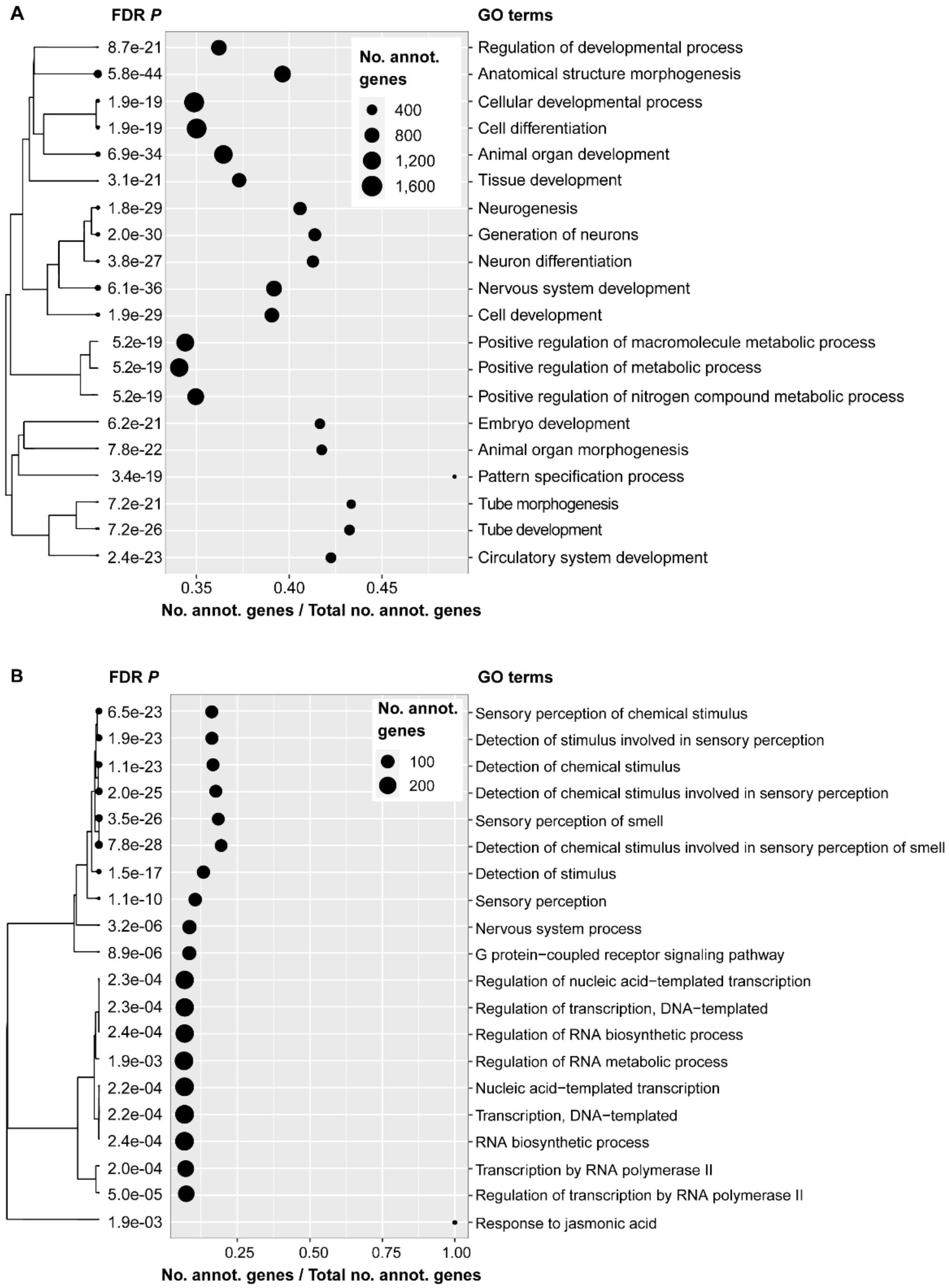

Fig. S12. Top 20 gene ontology (GO) terms enriched in (*A*) msHSBs and (*B*) EBRs.

Cladogram shows relationships between GO terms. Bigger dots at the cladogram tips indicate more significant false discovery rate corrected *P*-values (FDR *P*). Black bubble size depicts the number of genes annotated in each GO term in the analyzed list. The *x*-axis shows the ratio of genes annotated for each GO term in the analyzed list versus the background list (all protein-coding genes in the human genome).

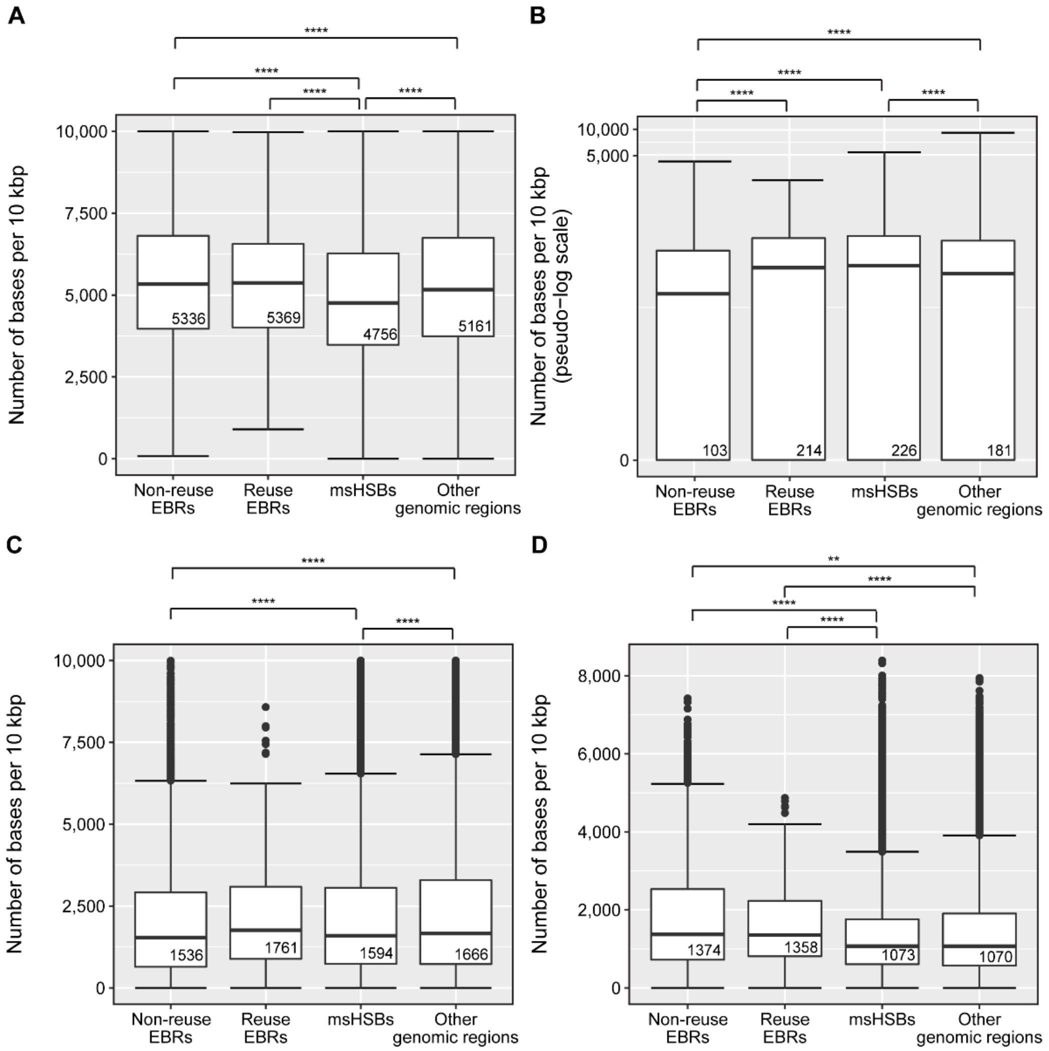

Fig. S13. Distribution of major classes of repetitive sequences within EBRs, msHSBs, and other regions of the human genome.

(*A*) Number of bases within all RepeatMasker annotated repeats in 10 kbp windows. (*B*) Number of bases within DNA retrotransposons in 10 kbp windows. (*C*) Number of bases within LINEs in 10 kbp windows. (*D*) Number of bases within SINEs in 10 kbp windows. Numbers within boxplots are group medians. Asterisks depict Bonferroni corrected *P*-values: **** $P \leq 0.0001$, *** $P \leq 0.001$, ** $P \leq 0.01$, * $P < 0.05$. Only significant comparisons are shown.

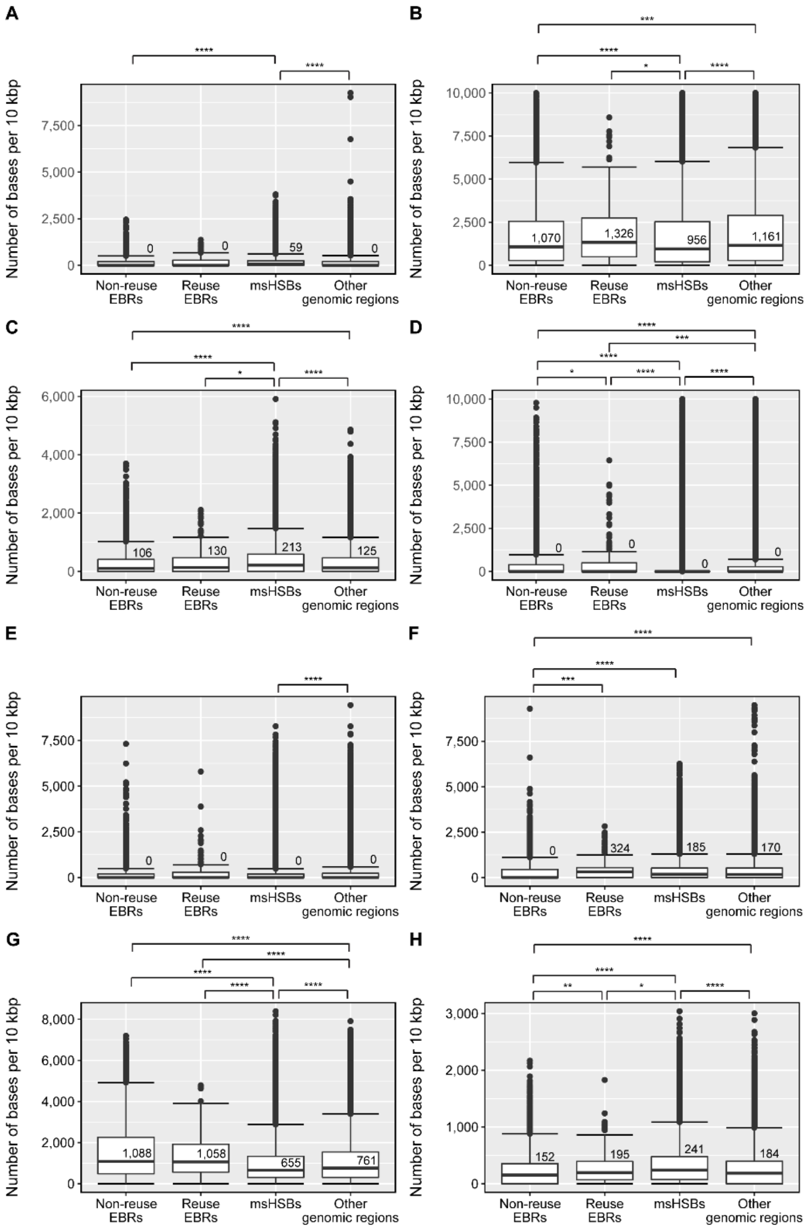

Fig. S14. Distribution of repeat subclasses within EBRs, msHSBs, and other regions of the human genome.

(*A*) Number of bases within DNA retrotransposons hAT-Charlie in 10 kbp windows. (*B*) Number of bases within L1 LINEs in 10 kbp windows. (*C*) Number of bases within L2 LINEs in 10 kbp windows. (*D*) Number of bases within ERV1 LTRs in 10 kbp windows. (*E*) Number of bases within ERVL LTRs in 10 kbp windows. (*F*) Number of bases within ERVL-MaLR LTRs in 10 kbp windows. (*G*) Number of bases within Alu SINEs in 10 kbp windows. (*H*) Number of bases within MIR SINEs in 10 kbp windows. Numbers within boxplots are group medians. Asterisks depict Bonferroni corrected *P*-values: **** $P \leq 0.0001$, *** $P \leq 0.001$, ** $P \leq 0.01$, * $P < 0.05$. Only significant comparisons are shown.

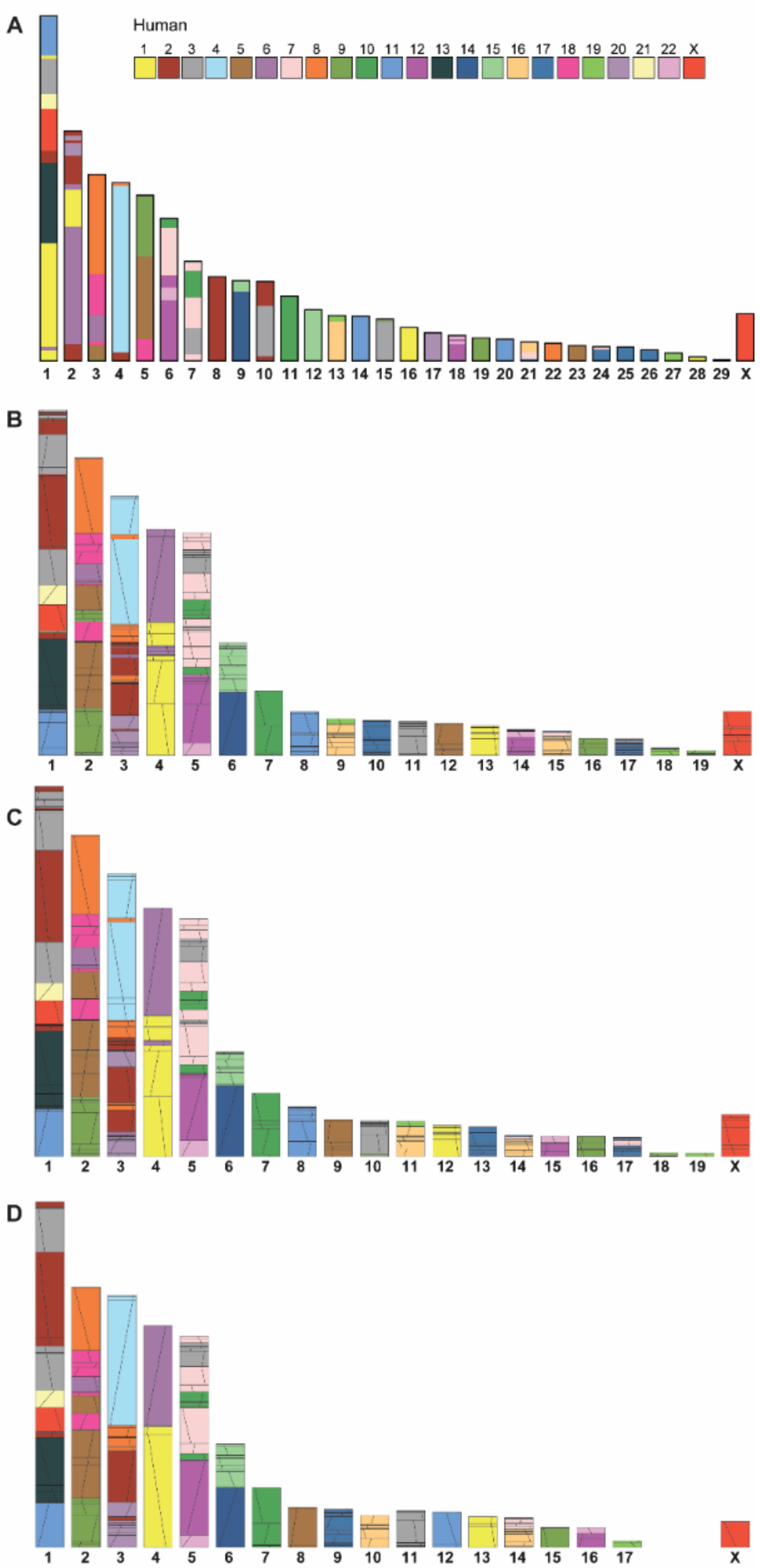
A
Human
1 2 3 4 5 6 7 8 9 10 11 12 13 14 15 16 17 18 19 20 21 22 X
1 2 3 4 5 6 7 8 9 10 11 12 13 14 15 16 17 18 19 20 21 22 23 24 25 26 27 28 29 X
B
1 2 3 4 5 6 7 8 9 10 11 12 13 14 15 16 17 18 19 X
C
1 2 3 4 5 6 7 8 9 10 11 12 13 14 15 16 17 18 19 X
D
1 2 3 4 5 6 7 8 9 10 11 12 13 14 15 16 17 X

Fig. S15. Comparison of reconstructed mammalian ancestor chromosomes.

(*A*) Mammalian ancestral chromosomes reconstructed by Zhou et al. (6). (*B*) Human genome-based reconstruction of mammalian ancestral chromosomes. (*C*) Sloth genome-based reconstruction of mammalian ancestral chromosomes. (*D*) Cattle genome-based reconstruction of mammalian ancestral chromosomes. Block colors indicate orthology to human chromosomes. For *B*, *C*, and *D*, lines within blocks depict same (left to right) or different (right to left) orientation compared to the respective human chromosomes.

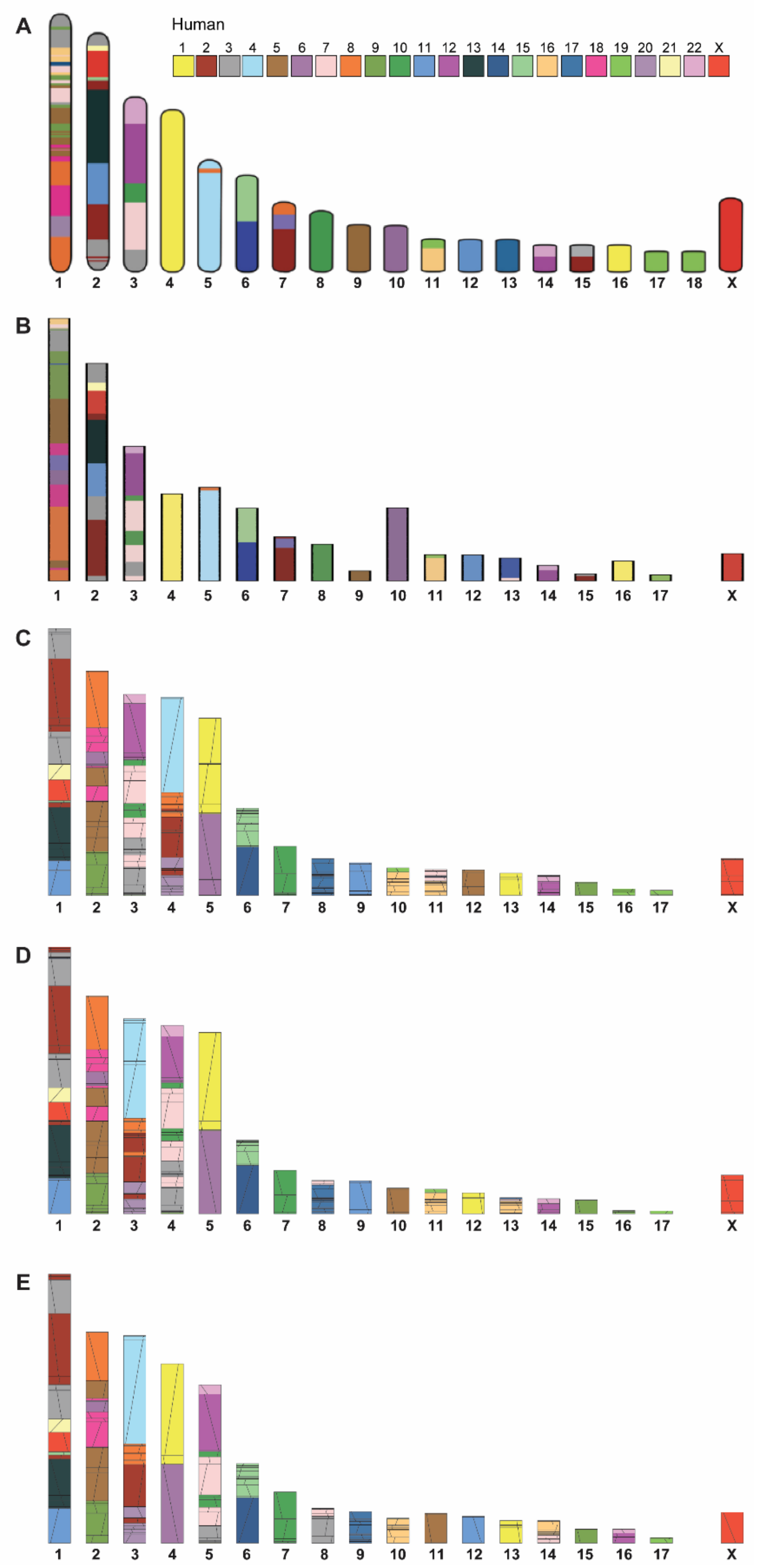
A
Human
1 2 3 4 5 6 7 8 9 10 11 12 13 14 15 16 17 18 19 20 21 22 X
1 2 3 4 5 6 7 8 9 10 11 12 13 14 15 16 17 18 X
B
1 2 3 4 5 6 7 8 9 10 11 12 13 14 15 16 17 X
C
1 2 3 4 5 6 7 8 9 10 11 12 13 14 15 16 17 X
D
1 2 3 4 5 6 7 8 9 10 11 12 13 14 15 16 17 X
E
1 2 3 4 5 6 7 8 9 10 11 12 13 14 15 16 17 X

Fig. S16. Comparison of reconstructed therian ancestor chromosomes.

(*A*) Therian ancestral chromosomes reconstructed by Deakin et al. (7). (*B*) Therian ancestral chromosomes reconstructed by Zhou et al. (6). (*C*) Human genome-based reconstruction of therian ancestral chromosomes. (*D*) Sloth genome-based reconstruction of therian ancestral chromosomes. (*E*) Cattle genome-based reconstruction of therian ancestral chromosomes. Block colors indicate orthology to human chromosomes. For *C*, *D*, and *E*, lines within blocks depict same (left to right) or different (right to left) orientation compared to the respective human chromosomes.

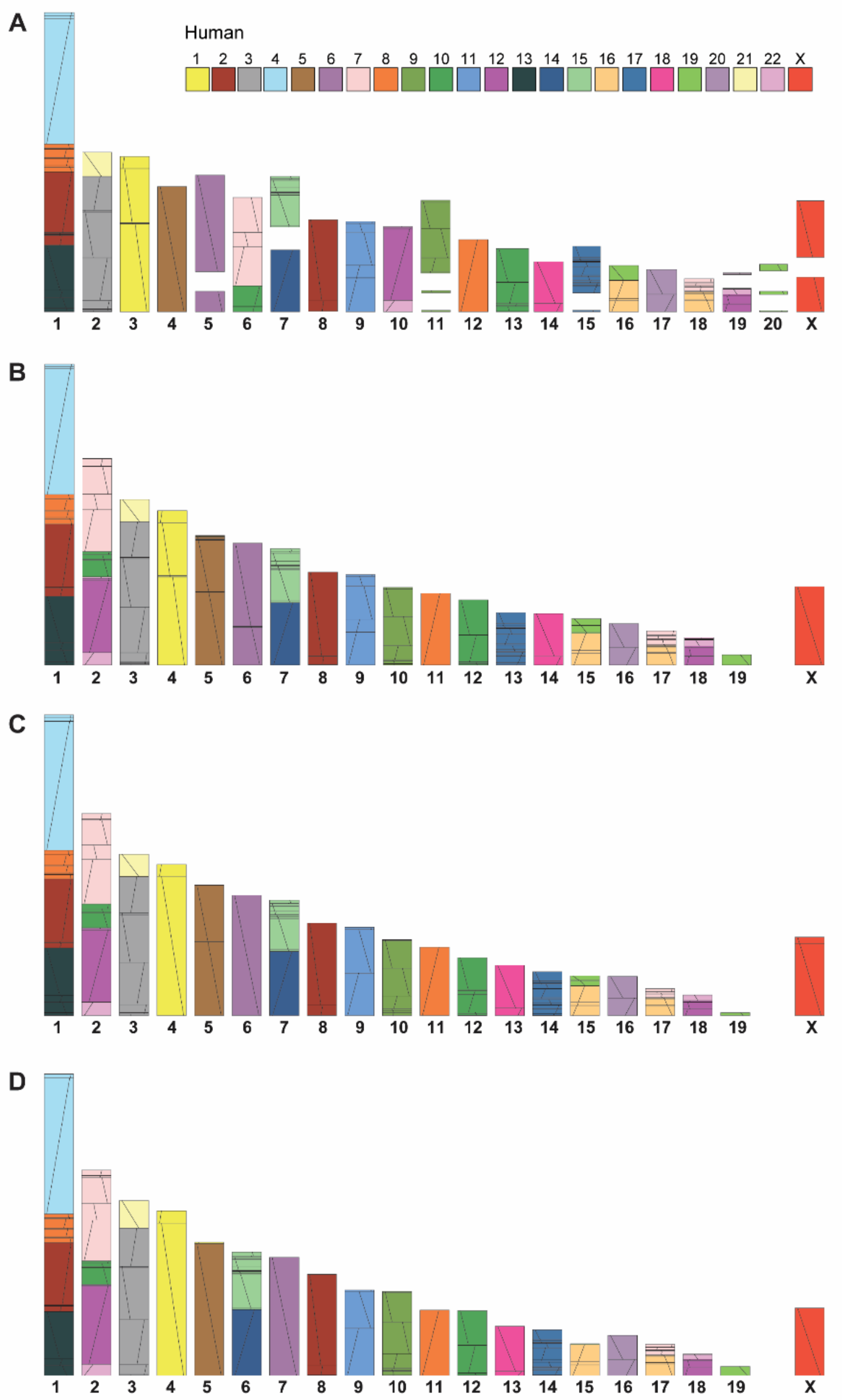
A
Human
1 2 3 4 5 6 7 8 9 10 11 12 13 14 15 16 17 18 19 20 21 22 X
1 2 3 4 5 6 7 8 9 10 11 12 13 14 15 16 17 18 19 20 X
B
1 2 3 4 5 6 7 8 9 10 11 12 13 14 15 16 17 18 19 X
C
1 2 3 4 5 6 7 8 9 10 11 12 13 14 15 16 17 18 19 X
D
1 2 3 4 5 6 7 8 9 10 11 12 13 14 15 16 17 18 19 X

Fig. S17. Comparison of reconstructed eutherian ancestor chromosomes.

(*A*) Eutherian ancestral chromosomes reconstructed by Kim et al. (3). (*B*) Human genome-based reconstruction or eutherian ancestral chromosomes. (*C*) Sloth genome-based reconstruction of eutherian ancestral chromosomes. (*D*) Cattle genome-based reconstruction of eutherian ancestral chromosomes. Block colors indicate orthology to human chromosomes. Lines within blocks depict same (left to right) or different (right to left) orientation compared to the respective human chromosomes.

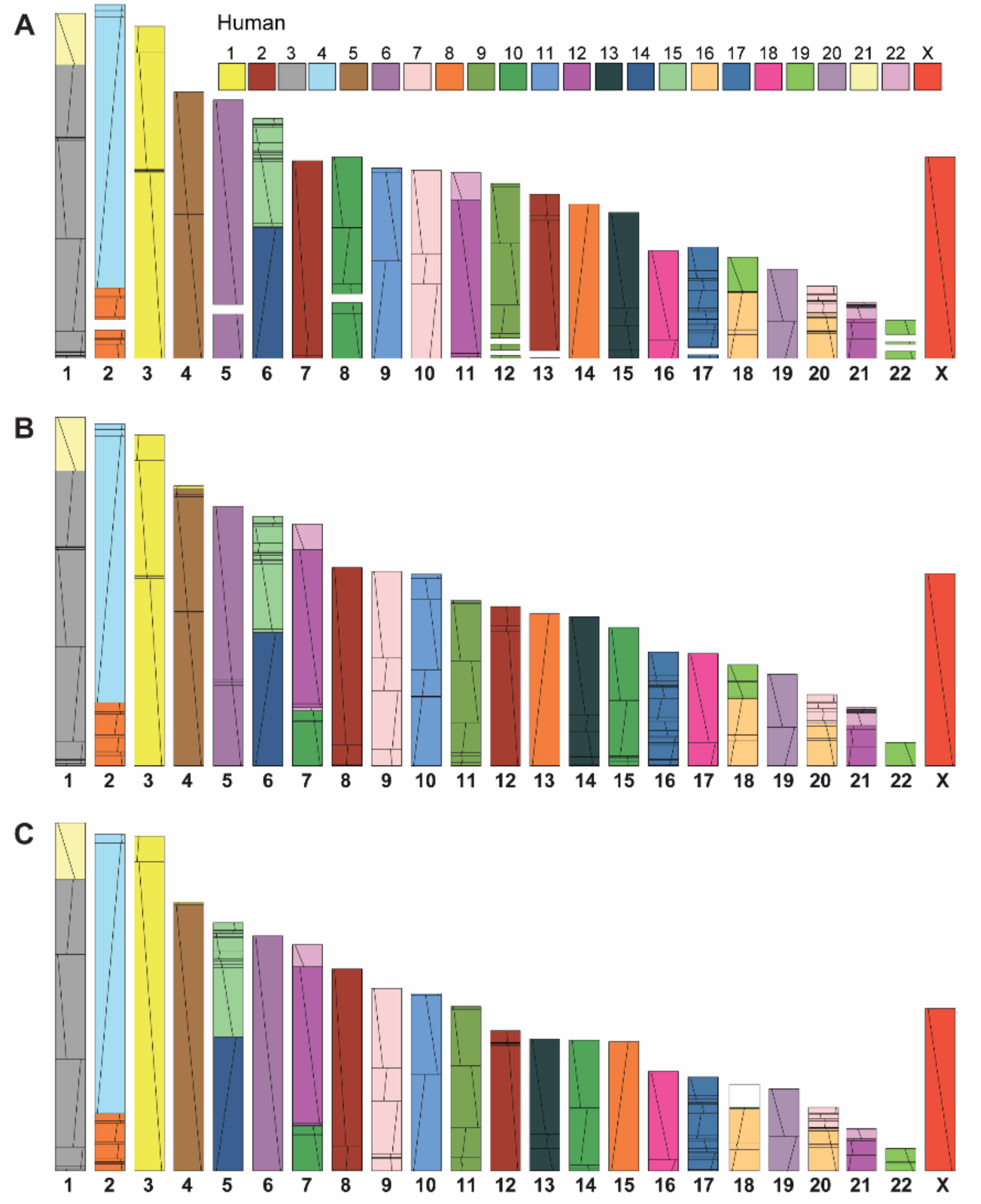
A
Human
1 2 3 4 5 6 7 8 9 10 11 12 13 14 15 16 17 18 19 20 21 22 X
1 2 3 4 5 6 7 8 9 10 11 12 13 14 15 16 17 18 19 20 21 22 X
B
1 2 3 4 5 6 7 8 9 10 11 12 13 14 15 16 17 18 19 20 21 22 X
C
1 2 3 4 5 6 7 8 9 10 11 12 13 14 15 16 17 18 19 20 21 22 X

Fig. S18. Comparison of reconstructed boreoeutherian ancestor chromosomes.

(*A*) Boreoeutherian ancestral chromosomes reconstructed by Kim et al. (3). (*B*) Human genome-based reconstruction of boreoeutherian ancestral chromosomes. (*C*) Sloth genome-based reconstruction of boreoeutherian ancestral chromosomes. (*D*) Cattle genome-based reconstruction of boreoeutherian ancestral chromosomes. Block colors indicate orthology to human chromosomes. Lines within blocks depict same (left to right) or different (right to left) orientation compared to the respective human chromosomes.

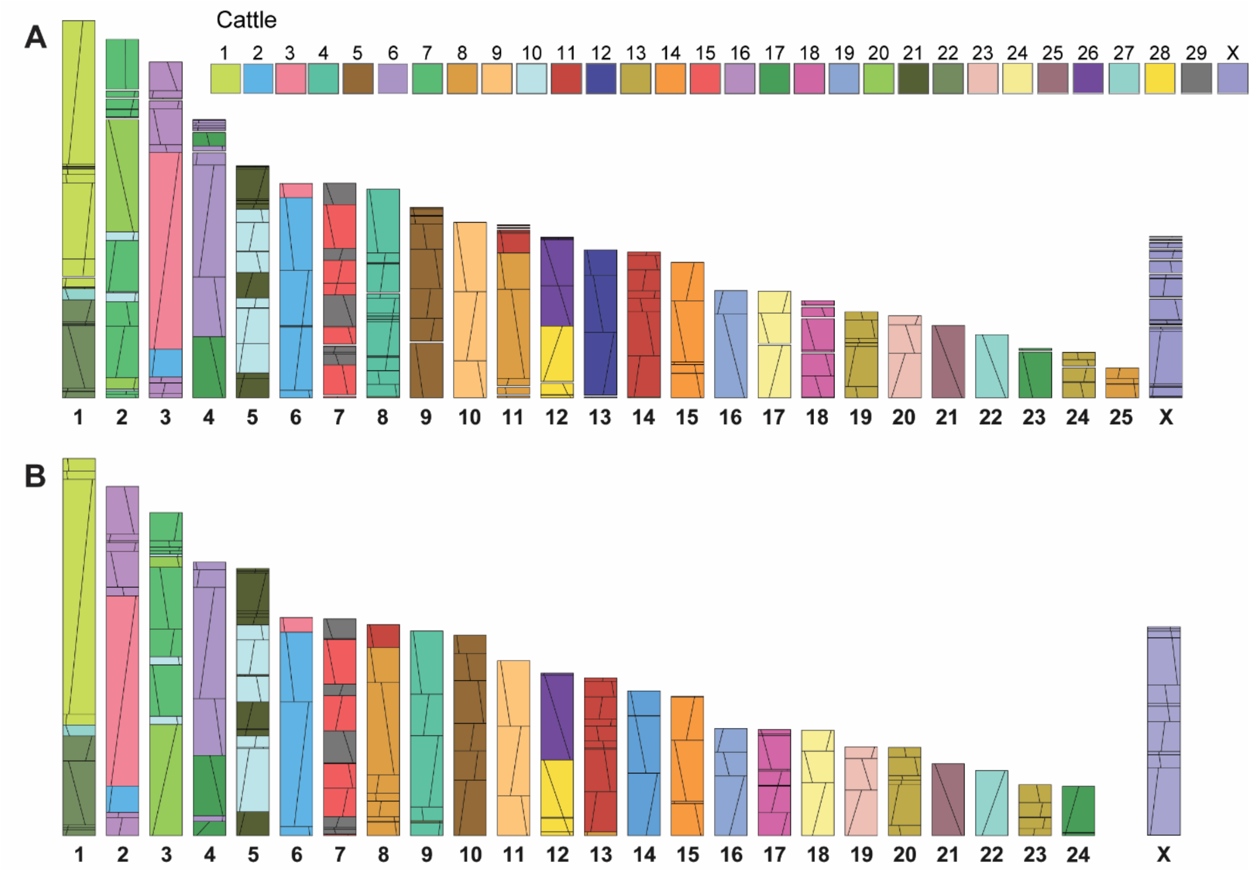

Fig. S19. Comparison of reconstructed cetartiodactyl ancestor chromosomes.

(*A*) Cetartiodactyl ancestral chromosomes reconstructed by Farré et al. (8). (*B*) Cattle genome-based reconstruction of ancestral cetartiodactyl chromosomes. Block colors indicate orthology to cattle chromosomes. Lines within blocks depict same (left to right) or different (right to left) orientation compared to the respective human chromosomes.

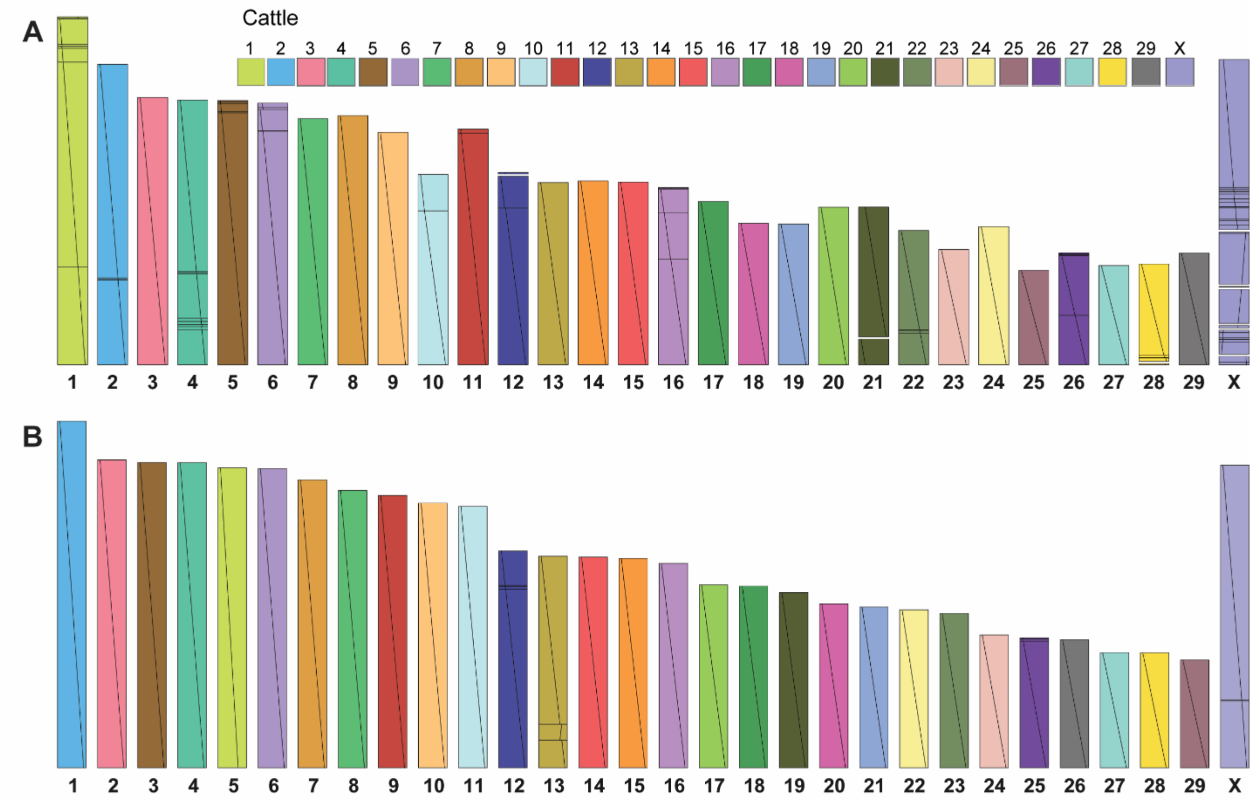

Fig. S20. Comparison of reconstructed ruminant (bovids) ancestor chromosomes.

(*A*) Ruminant ancestral chromosomes reconstructed by Farré et al. (8). (*B*) Cattle genome-based reconstruction of ruminant ancestral chromosomes. Block colors indicate orthology to cattle chromosomes. Lines within blocks depict same (left to right) or different (right to left) orientation compared to the respective human chromosomes.

Supplemental Tables

Table S1. Statistics of the reconstructed ancestral chromosomes.

Ref.[1]	Ancestor	Acronym	No. RACFs	Longest RACF (Mbp)	Total length RACFs (Mbp)	Coverage (%)[2]	No. reconstructed chromosomes	% in chrs.[3]
Human (hg38)	Mammalia	aMAM	74	273.05	2,645.73	87.29	19+X	99.23
	Theria	aTHE	63	182.50	2,687.18	88.66	17+X	99.85
	Eutheria	aEUT	26	381.33	2,755.11	90.90	19+X	100
	Boreoeutheria	aBOR	29	215.64	2,774.70	91.54	22+X	100
	Euarchontoglires	aEUA	31	225.54	2,807.04	92.61	23+X	100
	Euarchonta	aEUC	35	306.85	2,822.52	93.12	22+X	100
	Primatomorpha	aPMT	48	307.27	2,831.25	93.41	23+X	100
	Primata (hominidae)	aPRT	56	194.59	2,854.89	94.19	23+X	100
Cattle (bosTau9)	Mammalia	aMAM	41	206.22	2,306.69	87.76	17+X	99.85
	Theria	aTHE	39	237.53	2,451.61	93.27	17+X	100
	Eutheria	aEUT	32	274.48	2,528.21	96.19	19+X	100
	Boreoeutheria	aBOR	28	207.99	2,554.27	97.18	22+X	100
	Laurasiatheria	aLAU	30	212.37	2,563.03	97.51	23+X	100
	Scrotifera	aSCR	29	219.02	2,582.22	98.24	24+X	100
	Fereungulata	aFER	29	219.29	2,585.20	98.36	23+X	100
	Cetartiodactyla	aCET	37	221.70	2,596.88	98.80	24+X	99.95
	Cetruminantia	aCRU	46	205.73	2,600.78	98.95	24+X	100
	Ruminantia (bovids)	aRUM	35	185.77	2,567.54	97.68	29+X	100
Sloth (mChoDid1)	Mammalia	aMAM	64	328.76	2,722.39	86.97	19+X	99.25
	Theria	aTHE	60	181.29	2,811.84	89.83	17+X	99.90
	Eutheria	aEUT	25	408.98	2,914.12	93.10	19+X	100
	Atlantogenata	aATL	27	448.02	2,924.78	93.44	19+X	99.98
	Xenarthra	aXEN	54	262.98	2,981.33	95.25	24+X	99.88

[1] Reference genome; [2] Coverage of respective reference genome (3,088,269,832 bp for human-based reconstructions; 2,628,394,923 bp for cattle-based reconstructions; 3,130,157,497 bp for sloth-based reconstructions); [3] Percentage of reconstructed genome placed in chromosomes.

Table S2. Comparison of syntenic fragment adjacencies between human and sloth genome-based ancestral reconstructions.

	Human-based adjacencies in sloth-based reconstructions				Sloth-based adjacencies in human-based reconstructions			
**Ancestor[1]**	**No. adjacencies**	**Maintained**	**Extra**	**Inconsistent**	**No. adjacencies**	**Maintained**	**Extra**	**Inconsistent**
aMAM	237	153 (65%)	51 (22%)	33 (14%)	207	148 (71%)	18 (9%)	41 (20%)
aTHE	193	123 (64%)	46 (24%)	24 (12%)	164	123 (75%)	6 (4%)	35 (21%)
aEUT	124	88 (71%)	24 (19%)	12 (10%)	112	89 (79%)	16 (14%)	7 (6%)

[1] aEUT, eutherian ancestor; aTHE, therian ancestor; aMAM, mammalian ancestor.

Table S3. Comparison of syntenic fragment adjacencies between human and cattle genome-based ancestral reconstructions.

	Human-based adjacencies in cattle-based reconstructions				Cattle-based adjacencies in human-based reconstructions			
**Ancestor[1]**	**No. adjacencies**	**Maintained**	**Extra**	**Inconsistent**	**No. adjacencies**	**Maintained**	**Extra**	**Inconsistent**
aMAM	237	92 (39%)	74 (31%)	71 (30%)	134	65 (49%)	18 (13%)	51 (38%)
aTHE	193	107 (55%)	45 (23%)	41 (21%)	146	93 (64%)	12 (8%)	41 (28%)
aEUT	124	88 (71%)	24 (19%)	12 (10%)	101	86 (85%)	0 (0%)	15 (15%)
aBOR	123	83 (67%)	29 (24%)	11 (9%)	106	82 (77%)	3 (3%)	21 (20%)

[1] aBOR, boreoeutherian ancestor; aEUT, eutherian ancestor; aTHE, therian ancestor; aMAM, mammalian ancestor.

Table S4. Statistics of ancestral chromosome regions with structural differences in reconstructions depending on the reference genome used.

Anc.[1]	Ref.[2]	No. of regions	Total length (kbp)	Average length (kbp)	Median length (kbp)	Percent of reconstructed genome length
aBOR	*Human versus cattle genome-based reconstructions*					
	Human	10	10,479	1,048	443	0.41
	Cattle		9,676	968	420	0.41
aEUT	*Human versus cattle genome-based reconstructions*					
	Human	15	7,466	498	474	0.30
	Cattle		7,222	481	470	0.32
	Human versus sloth genome-based reconstructions					
	Human	11	9,914	901	467	0.40
	Sloth		10,342	940	510	0.41
aTHE	*Human versus cattle genome-based reconstructions*					
	Human	36	227,995	6,514	2,120	9.78
	Cattle		200,251	5,721	1,768	10.13
	Human versus sloth genome-based reconstructions					
	Human	22	97,138	4,415	1,912	4.17
	Sloth		102,065	4,639	1,899	4.41
aMAM	*Human versus cattle genome-based reconstruction*					
	Human	69	422,894	6,129	1,927	18.78
	Cattle		372,949	5,405	1,582	21.16
	Human versus sloth genome-based reconstruction					
	Human	27	194,633	7,209	1,258	8.65
	Sloth		215,501	7,981	1,333	9.72

[1] Ancestor; aBOR, boreoeutherian ancestor; aEUT, eutherian ancestor; aTHE, therian ancestor; aMAM, mammalian ancestor. [2] Reference genome.

Table S5. Recovery of complete BUSCOs common to the human and platypus genomes (*n*=6,722) in the reconstructed mammalian ancestor chromosomes using the human, cattle, and sloth genomes as a reference.

Reference genome	Present (%)	Absent (%)
**Human**	6,669 (99%)	53 (1%)
**Cattle**	6,039 (96%)	266 (4%)
**Sloth**	5,924 (96%)	229 (4%)

Table S6. Number of evolutionary breakpoint regions (EBRs), breakpoint rates (breakpoints/My) and chromosome rearrangements that occurred during mammalian evolution.

Lineage	Branch[1]	Branch length (My)	My from present	No. EBRs	Breakpoint rate*	No. rearrangements: Inversions	Fissions	Fusions	Total
Human (hg38)	aMAM → aTHE	18	177	72	3.93 ↑	90 ↑	3	3	96 ↑
	aTHE → aEUT	53	159	102	1.92	94	16	14 ↑	124
	aEUT → aBOR	9	106	1	0.11 ↓	1 ↓	3 ↓	0 ↓	4
	aBOR → aEUA	7	97	7	1.05	4	1	0 ↓	5
	aEUA → aEUC	8	90	6	0.78	9	1	2 ↑	12
	aEUC → aPMT	6	82	16	2.50	24 ↑	2 ↑	1	27 ↑
	aPMT → aPRT	69	76	97	1.40	73	4	4	81
	aPRT → Human	7	7	22	3.31 ↑	15	0 ↓	1	16
	**Total**			**323**		**310**	**30**	**25**	**365**
Sloth (mChoDid1)	aMAM → aTHE	18	177	69	3.76	84	5	6	95
	aTHE → aEUT	53	159	98	1.84	73	14	13	100
	aEUT → aATL	5	106	1	0.20	4	0	0	4
	aATL → aXEN	35	101	32	0.92	26	8	2	36
	aXEN → Sloth	66	66	62	0.94	39	6	6	51
	**Total**			**262**		**226**	**33**	**28**	**286**
Cattle (bosTau9)	aMAM → aTHE	18	177	7	0.38	16	1	1	18
	aTHE → aEUT	53	159	57	1.07	60	15	13	88
	aEUT → aBOR	9	106	1	0.11 ↓	4 ↓	3	0	7
	aBOR → aLAU	7	97	5	0.70	7	2	1	10
	aLAU → aSCR	11	90	7	0.65	6	2	1	9
	aSCR → aFER	1	79	1	1.29	2	1 ↑	2 ↑	5 ↑
	aFER → aCET	16	78	35	2.22	22	4	4	30
	aCET → aCRU	6	62	23	3.83 ↑	21 ↑	1	1	23
	aCRU → aRUM	31	56	112	3.57 ↑	87 ↑	13	7	107
	aRUM → Cattle	25	25	9	0.37	6 ↓	0 ↓	0	6
	**Total**			**257**		**231**	**42**	**30**	**303**

* Average breakpoint rates from the mammalian ancestor to the human, sloth and cattle genomes are 1.88, 1.54, and 1.46 breakpoints/My, respectively.

↑ Significantly higher than average across all branches for respective lineage (FDR corrected *P*<0.05).

↓ Significantly lower than average across all branches for respective lineage (FDR corrected *P*<0.05).

[1] aATL, Atlantogenata ancestor; aBOR, boreoeutherian ancestor; aCET, Cetartiodactyla ancestor; aCRU, Cetruminantia ancestor; aEUA, Euarchontoglires ancestor; aEUC, Euarchonta ancestor; aEUT, eutherian ancestor; aFER, Fereungulata ancestor; aLAU, laurasiatherian ancestor; aMAM, mammalian ancestor; aPMT; Primatomorpha ancestor; aPRT, primates (Hominidae) ancestor; aRUM, Ruminantia (bovids) ancestor; aSCR, Scrotifera ancestor; aTHE, therian ancestor; aXEN, Xenarthra ancestor.

Table S7. Ancestral syntenies identified in the reconstructed ancestors at 1 Mbp resolution.

Common ancestors for all three lineages			Common to human and cattle lineage	Human lineage			Sloth lineage		Cattle lineage					
**aMAM**	**aTHE**	**aEUT**	**aBOR**	**aEUA**	**aEUC**	**aPMT**	**aATL**	**aXEN**	**aLAU**	**aSCR**	**aFER**	**aCET**	**aCRU**	**aRUM**
12q-22	12q-22	12q-22	12q-22	12q-22	22-12q-22	12q-22	12q-22	12q-22	12q-22	12q-22	12q-22-**18**	12q-22	12q-22	4q-12q-22
22-12pq-22-**10p**-7pq-3pq-7pq-**10p**-7pq-3pq-7pq	22-12pq-22-**10p**-7pq-**10p**-7pq-3pq-7pq-3pq-9q	22-12pq-22-10p-7pq	10p-22-12pq-22	22-12pq-22	22-12pq-22	22-12pq-22	22-12pq-22-**10p**-7p	22-12pq-22-**10p**	22-12pq	22-12pq	22-12pq	22-12pq	22-12pq	12pq-22-12pq-22
11pq-**13**-2q-15q-Xp-**21**-3pq-2q-3pq-2q-3pq-2q-3pq	11pq-**13**-2q-15q-Xp-**21**-3pq-2q-3pq	**3-21**	**3-21**	**3-21**	**3-21**	**3-21**	**3-21**	**3-21**	**3-21**	**3-21**	**3-21**	**3-21**	**3-21**	**3-21-3**
**20**-2pq-**20**-2pq-**8p**-2pq-6p-2pq-**8p-4-8p-4**	**20**-2pq-**20**-2pq-**8p**-2pq-**8p-4**	**13**-2pq-**8p-4**	**8p-4**	**8p-4**	**8-4**	**8-4**	**13**-2pq-**8p-4**	**4-8p**-2p	**8p**-4pq-**8p**-4pq	8p-4pq	8p-4pq	4pq-8p-**9**	**9**-8p-4pq-8p-**9**	4pq-9pq-8p-9pq
**16q**-19q	**16q**-19q	**16q-19q**	**16q-19q**	**16q-19q**	**16q-19q**	**16q-19q**	**16q-19q**	**16q-19q**	**16q-19q**	**16q-19q**	**16q-19q**	**16q-19q**	**16q-19q**	**16q-19q**
**16p**-7p-17p	**16p**-7pq-17p-7pq	**16p**-7pq	**16p**-7pq	**16p**-7pq	**16p**-7pq		**16p**-7pq	**16p**-7pq	**16p**-7pq	**16p**-7pq	**16p**-7pq	**16p**-7pq	**20-16p**-7pq	**16p**-7pq
**14**-15	**14**-15	**14**-15	**14**-15	**14**-15			**14**-15	**14**-15	**14**-15	**14**-15	**14**-15	**14**-15	**14**-15	14-15
1pq-6pq-1pq-6pq	1pq-6pq							**1**-6q						
9pq-5pq-**18**-9pq-5pq-**18**-6p-**18-8q**	9pq-5pq-**18**-5pq-**18**-6p-**18-8q**													
17pq-7q-17pq														
9q-3p														
		**5**-1q	**5**-1q	**5**-1q	**5**-1q		**5**-1q		**5**-1q	**5**-1q	**5**-1q	**5**-1q-**19p**-1q	**5**-1q-**19p**-1q	5q-**19p**-5q
								10p-7pq						
								5q-6q						
										8p-4q-8p	8p-4q-8p	8p-4q-8p	8p-4q-8p	8p-4q-8p-3p
											1q-**10q**	1q-10q	1q-**10q**	1q-10q
													15q-2q	
														**20-10p-20-10p-20**
														2p-1p
														2q-1pq
														2pq-9q
														5q-15-14-15-14-15-14

Ancestral syntenies are reported as associations of human chromosomes. Shaded ancestor abbreviations and full shaded columns depict ancestral chromosomes reported in this work for the first time to our knowledge. Other shaded cells depict ancestral chromosomes with newly identified ancestral syntenies. Non-shaded cells depict chromosomes with ancestral syntenies previously reported (3, 7-12). Primate (Hominidae) ancestor not shown because its reconstructed chromosomes are the same as those of human. aATL, Atlantogenata ancestor; aBOR, boreoeutherian ancestor; aCET, Cetartiodactyla ancestor; aCRU, Cetruminantia ancestor; aEUA, Euarchontoglires ancestor; aEUC, Euarchonta ancestor; aEUT, eutherian ancestor; aFER, Fereungulata ancestor; aLAU, laurasiatherian ancestor; aMAM, mammalian ancestor; aPRT, primates (Hominidae) ancestor; aRUM, Ruminantia (bovids) ancestor; aSCR, Scrotifera ancestor; aTHE, therian ancestor; aXEN, Xenarthra ancestor.

Table S8: Length distribution of evolutionary breakpoint regions (EBRs) in each of the studied lineages.

Lineage	No. EBRs	Median	Mean	Max.
**Human**	323	110 kbp	621 kbp	28 Mbp
**Sloth**	262	281 kbp	728 kbp	18 Mbp
**Cattle**	257	348 bp	244 kbp	4 Mbp

EBR length was calculated based on the EBR coordinates on the reference species genome for each lineage.

Table S9. Distribution of human protein-coding genes in the mammalian ancestor chromosomes (MAMs).

MAMs	Length (Mbp)	Density per Mbp	Fraction within (%)	Average length (bp)
1	411	6	43 ↓	94,908 ↑
2	354	4 ↓	40 ↓	105,644 ↑
3	308	6	44 ↓	87,067 ↑
4	269	8	48 ↓	71,692
5	265	6	52	102,584 ↑
6	133	8	52	75,164
7	76	7	54	86,550 ↑
8	52	14	44 ↓	44,797 ↓
9	44	11	47 ↓	51,240
10	42	18	55	36,513 ↓
11	41	7	62 ↑	100,715 ↑
12	38	10	51	73,204
13	35	14	58	45,020 ↓
14	31	11	61 ↑	62,422
15	29	15	62 ↑	10,294 ↓
16	20	14	61 ↑	51,398
17	20	15	70 ↑	51,166
18	9	52 ↑	74 ↑	26,281 ↓
19	6	64 ↑	70 ↑	22,720 ↓
X	52	10	32 ↓	55,294
**Average**		**15**	**54**	**62,734**

↑ Significantly higher than average across all MAMs (FDR corrected $P<0.05$).

↓ Significantly lower than average across all MAMs (FDR corrected $P<0.05$).

Table S10. Summary statistics for the identified mammalian multispecies homologous synteny blocks (msHSBs) based of their coordinates in the human genome.

	Longer than 300 kbp	Longer than 1 Mbp
**Total no. msHSBs**	1,215	522
**Total msHSB length (bp)**	1,690,855,716	1,343,458,458
**Human genome coverage (%)**	55	44
**Median msHSB length (bp)**	849,095	1,952,510
**Mean msHSB length (bp)**	1,391,651	2,573,675
**Expected max. length (bp)**	8,821,980	
**Observed max. length (bp)**	22,111,330	
**No. msHSBs longer than expected (*P*<0.05)**	5	

Table S11: Median [interquartile range] length of genes (kbp) within msHSBs, EBRs, and other regions of the human genome.

Species	*n*	msHSB	EBR	Rest of the genome
**Human**	19,878	36 [13 - 94]	16 [6 - 35]	23 [8 - 59]
**Cattle**	15,310	28 [10 - 76]	12 [6 - 28]	18 [7 - 49]
**Greater horseshoe bat**	15,520	25 [8 - 66]	11 [4 - 24]	17 [6 - 44]
**African elephant**	14,838	23 [7 - 61]	10 [4 - 26]	15 [5 - 42]
**Hoffmann's two-fingered sloth**	9,513	22 [8 - 46]	9 [4 - 21]	16 [6 - 36]
**Chicken**	12,015	16 [6 - 40]	10 [5 - 21]	14 [6 - 34]

For each species comparison, all groups were statistically different ($P<0.001$) by pairwise comparisons using Wilcoxon rank sum test with continuity correction.

For human, all protein-coding genes were used. For the remaining species length distribution was calculated using only 1:1 orthologs to human protein-coding genes.

Table S12: Statistics for major classes of repeats within msHSBs, reuse and non-reuse EBRs, and other regions of the human genome.

	Group	Min.	1st quartile	Median	3rd quartile	Max.	Mean	SD
**All repeats**	Non-reuse EBRs	0	4,050	5,530	7,243	10,000	5,686	2,311
	Reuse EBRs	898	4,010	5,369	6,563	9,975	5,400	1,819
	msHSBs	0	3,480	4,756	6,269	10,000	4,938	1,991
	Other regions	0	3,740	5,161	6,746	10,000	5,283	2,117
**DNA**	Non-reuse EBRs	0	0	103	345	4,144	255	411
	Reuse EBRs	0	0	214	488	2,458	332	384
	msHSBs	0	0	226	518	5,353	378	488
	Other regions	0	0	181	454	9,252	330	467
**LINE**	Non-reuse EBRs	0	548	1,533	3,005	9,993	2,066	1,968
	Reuse EBRs	0	887	1,761	3,089	8,576	2,144	1,697
	msHSBs	0	735	1,594	3,059	10,000	2,186	1,959
	Other regions	0	728	1,666	3,292	10,000	2,304	2,088
**SINE**	Non-reuse EBRs	0	532	1,155	2,136	7,421	1,502	1,335
	Reuse EBRs	0	811	1,358	2,231	4,870	1,602	1,040
	msHSBs	0	608	1,073	1,761	8,392	1,340	1,052
	Other regions	0	572	1,070	1,907	7,943	1,400	1,177
**LTR**	Non-reuse EBRs	0	0	513	1,323	9,999	1,002	1,404
	Reuse EBRs	0	349	826	1,572	7,450	1,156	1,269
	msHSBs	0	0	501	1,166	10,000	865	1,136
	Other regions	0	54	583	1,347	10,000	999	1,285
**Segmental duplications**	Non-reuse EBRs	0	0	0	10,000	10,000	4,164	4,792
	Reuse EBRs	0	0	0	2,230	10,000	2,282	4,022
	msHSBs	0	0	0	0	10,000	129	988
	Other regions	0	0	0	0	10,000	832	2,635

Table S13: Statistics for subclasses of repeats within msHSBs, reuse and non-reuse EBRs, and other regions of the human genome.

	Group	Min.	1st quartile	Median	3rd quartile	Max.	Mean	SD
DNA hAT-Charlie	Non-reuse EBRs	0	0	0	203	2,455	138	236
	Reuse EBRs	0	0	0	272	1,369	169	248
	msHSBs	0	0	59	244	3,811	171	268
	Other regions	0	0	0	210	9,252	147	255
LINE L1	Non-reuse EBRs	0	284	1,070	2,554	9,993	1,757	1,956
	Reuse EBRs	0	494	1,326	2,746	8,576	1,810	1,717
	msHSBs	0	204	956	2,528	10,000	1,722	2,010
	Other regions	0	286	1,161	2,901	10,000	1,936	2,139
LINE L2	Non-reuse EBRs	0	0	106	411	3,702	287	429
	Reuse EBRs	0	0	130	469	2,110	306	422
	msHSBs	0	0	213	591	5,913	397	501
	Other regions	0	0	125	466	4,863	319	459
LTR ERV1	Non-reuse EBRs	0	0	0	389	9,778	395	1,022
	Reuse EBRs	0	0	0	493	6,442	427	897
	msHSBs	0	0	0	0	9,999	244	772
	Other regions	0	0	0	278	10,000	341	935
LTR ERVL	Non-reuse EBRs	0	0	0	199	7,316	184	444
	Reuse EBRs	0	0	0	288	5,797	244	603
	msHSBs	0	0	0	195	8,279	191	497
	Other regions	0	0	0	234	9,432	208	526
LTR ERVL-MaLR	Non-reuse EBRs	0	0	0	445	9,292	337	572
	Reuse EBRs	0	0	324	541	2,822	414	530
	msHSBs	0	0	185	519	6,266	388	578
	Other regions	0	0	170	518	9,490	392	605
SINE MIR	Non-reuse EBRs	0	0	152	352	2,168	233	286
	Reuse EBRs	0	68	195	392	1,828	268	276
	msHSBs	0	72	241	477	3,039	323	320
	Other regions	0	0	184	395	3,002	261	297
SINE Alu	Non-reuse EBRs	0	485	1,088	2,258	7,198	1,539	1,404
	Reuse EBRs	0	563	1,058	1,920	4,790	1,330	1,044
	msHSBs	0	304	655	1,334	8,392	1,012	1,026
	Other regions	0	306	761	1,543	7,911	1,137	1,146

Table S14: Comparison of breakpoint rate estimates (breakpoints/My) to previous reports.

	This paper	Murphy et al. (2005)	Kim et al. (2017)	Farré et al. (2019)
**Human lineage**				
aEUT → aBOR	0.1	-	0.8	-
aBOR → aEUA	1.0	-	1.4	-
aPRT → Human	3.3	-	2.0	-
aEUT → Human	1.5	-	1.8	-
**Cattle lineage**				
aBOR → aFER	0.9	0.1	-	-
aFER → aCET	2.2	0.4	-	1.7
aCET → aRUM	3.8	-	-	2.8
aCET → Cattle	2.6	1.1	-	4.1

aBOR, boreoeutherian ancestor; aCET, Cetartiodactyla ancestor; aEUA, Euarchontoglires ancestor; aEUT, eutherian ancestor; aFER, Fereungulata ancestor; aPRT, primates (Hominidae) ancestor; aRUM, Ruminantia (bovids) ancestor.

Legends for Dataset S1 to S14

Dataset S1 (separate file). Statistics for the genome assemblies of descendant and outgroup species.

Dataset S2 (separate file). Genome alignment coverage statistics.

Dataset S3 (separate file). Manually curated RACFs for human genome-based reconstructed ancestors.

Dataset S4 (separate file). Manually curated RACFs for cattle genome-based reconstructed ancestors.

Dataset S5 (separate file). Manually RACFs for sloth genome-based reconstructed ancestors.

Dataset S6 (separate file). Syntenic fragment coverage of the reconstructed mammalian ancestor, descendant, and outgroups species' genomes.

Dataset S7 (separate file). Evolutionary breakpoint regions identified in the human lineage.

Dataset S8 (separate file). Evolutionary breakpoint regions identified in the sloth lineage.

Dataset S9 (separate file). Evolutionary breakpoint regions identified in the cattle lineage.

Dataset S10 (separate file). Mammalian multispecies homologous synteny blocks longer than 300 Kbp as identified in the human genome.

Dataset S11 (separate file). Gene ontology terms enriched in mammalian multispecies homologous synteny blocks and evolutionary breakpoint regions identified in the human lineage.

Dataset S12 (separate file). Orthology maps for each pairwise comparison on the human lineage.

Dataset S13 (separate file). Orthology maps for each pairwise comparison on the sloth lineage.

Dataset S14 (separate file). Orthology maps for each pairwise comparison on the cattle lineage.

SI References

1. Harris RS (2007) Improved pairwise alignment of genomic DNA. Ph.D. (The Pennsylvania State University).
2. Kent WJ, Baertsch R, Hinrichs A, Miller W, & Haussler D (2003) Evolution's cauldron: duplication, deletion, and rearrangement in the mouse and human genomes. *Proceedings of the National Academy of Sciences* 100(20):11484-11489.
3. Kim J*, et al.* (2017) Reconstruction and evolutionary history of eutherian chromosomes. *Proceedings of the National Academy of Sciences* 114(27):E5379-E5388.
4. Damas J, Kim J, Farré M, Griffin DK, & Larkin DM (2018) Reconstruction of avian ancestral karyotypes reveals differences in the evolutionary history of macro- and microchromosomes. *Genome Biology* 19(1):155.
5. Manni M, Berkeley MR, Seppey M, Simão FA, & Zdobnov EM (2021) BUSCO update: novel and streamlined workflows along with broader and deeper phylogenetic coverage for scoring of eukaryotic, prokaryotic, and viral genomes. *Molecular Biology and Evolution* 83(10):4647-4654.
6. Zhou Y*, et al.* (2021) Platypus and echidna genomes reveal mammalian biology and evolution. *Nature* 592(7856):756-762.
7. Deakin JE*, et al.* (2013) Reconstruction of the ancestral marsupial karyotype from comparative gene maps. *BMC evolutionary biology* 13(1):258.
8. Farré M*, et al.* (2019) Evolution of gene regulation in ruminants differs between evolutionary breakpoint regions and homologous synteny blocks. *Genome Research* 29(4):576-589.
9. Ruiz-Herrera A, Farre M, & Robinson TJ (2012) Molecular cytogenetic and genomic insights into chromosomal evolution. *Heredity* 108(1):28-36.
10. Froenicke L (2005) Origins of primate chromosomes - as delineated by Zoo-FISH and alignments of human and mouse draft genome sequences. *Cytogenetic and genome research* 108(1-3):122-138.
11. Azevedo NF*, et al.* (2012) Chromosome painting in three-toed sloths: a cytogenetic signature and ancestral karyotype for Xenarthra. *BMC evolutionary biology* 12:36.
12. Kulemzina AI*, et al.* (2011) Chromosome painting in Tragulidae facilitates the reconstruction of Ruminantia ancestral karyotype. *Chromosome Research* 19(4):531-539.

Zoonomia authors list

Gregory Andrews[1], Joel C. Armstrong[2], Matteo Bianchi[3], Bruce W. Birren[4], Kevin R. Bredemeyer[5], Ana M. Breit[6], Matthew J. Christmas[3], Hiram Clawson[2], Joana Damas[7], Federica Di Palma[8,9], Mark Diekhans[2], Michael X. Dong[3], Eduardo Eizirik[10], Kaili Fan[1], Cornelia Fanter[11], Nicole M. Foley[5], Karin Forsberg-Nilsson[12,13], Carlos J. Garcia[14], John Gatesy[15], Steven Gazal[16], Diane P. Genereux[4], Linda Goodman[17], Jenna Grimshaw[14], Michaela K. Halsey[14], Andrew J. Harris[5], Glenn Hickey[18], Michael Hiller[19,20,21], Allyson G. Hindle[11], Robert M. Hubley[22], Graham M. Hughes[23], Jeremy Johnson[4], David Juan[24], Irene M. Kaplow[25,26], Elinor K. Karlsson[1,4,27], Kathleen C. Keough[17,28,29], Bogdan Kirilenko[19,20,21], Klaus-Peter Koepfli[30,31,32], Jennifer M. Korstian[14], Amanda Kowalczyk[25,26], Sergey V. Kozyrev[3], Alyssa J. Lawler[4,26,33], Colleen Lawless[23], Thomas Lehmann[34], Danielle L. Levesque[6], Harris A. Lewin[7,35,36], Xue Li[1,4,37], Abigail Lind[28,29], Kerstin Lindblad-Toh[3,4], Ava Mackay-Smith[38], Voichita D. Marinescu[3], Tomas Marques-Bonet[39,40,41,42], Victor C. Mason[43], Jennifer R. S. Meadows[3], Wynn K. Meyer[44], Jill E. Moore[1], Lucas R. Moreira[1,4], Diana D. Moreno-Santillan[14], Kathleen M. Morrill[1,4,37], Gerard Muntané[24], William J. Murphy[5], Arcadi Navarro[39,41,45,46], Martin Nweeia[47,48,49,50], Sylvia Ortmann[51], Austin Osmanski[14], Benedict Paten[2], Nicole S. Paulat[14], Andreas R. Pfenning[25,26], BaDoi N. Phan[25,26,52], Katherine S. Pollard[28,29,53], Henry E. Pratt[1], David A. Ray[14], Steven K. Reilly[38], Jeb R. Rosen[22], Irina Ruf[54], Louise Ryan[23], Oliver A. Ryder[55,56], Pardis C. Sabeti[4,57,58], Daniel E. Schäffer[25], Aitor Serres[24], Beth Shapiro[59,60], Arian F. A. Smit[22], Mark Springer[61], Chaitanya Srinivasan[25], Cynthia Steiner[55], Jessica M. Storer[22], Kevin A. M. Sullivan[14], Patrick F. Sullivan[62,63], Elisabeth Sundström[3], Megan A. Supple[59], Ross Swofford[4], Joy-El Talbot[64], Emma Teeling[23], Jason Turner-Maier[4], Alejandro Valenzuela[24], Franziska Wagner[65], Ola Wallerman[3], Chao Wang[3], Juehan Wang[16], Zhiping Weng[1], Aryn P. Wilder[55], Morgan E. Wirthlin[25,26,66], James R. Xue[4,57], Xiaomeng Zhang[4,25,26]

Affiliations:
[1]Program in Bioinformatics and Integrative Biology, UMass Chan Medical School; Worcester, MA 01605, USA.
[2]Genomics Institute, University of California Santa Cruz; Santa Cruz, CA 95064, USA.
[3]Department of Medical Biochemistry and Microbiology, Science for Life Laboratory, Uppsala University; Uppsala, 751 32, Sweden.
[4]Broad Institute of MIT and Harvard; Cambridge, MA 02139, USA.
[5]Veterinary Integrative Biosciences, Texas A&M University; College Station, TX 77843, USA.
[6]School of Biology and Ecology, University of Maine; Orono, ME 04469, USA.
[7]The Genome Center, University of California Davis; Davis, CA 95616, USA.
[8]Genome British Columbia; Vancouver, BC, Canada.
[9]School of Biological Sciences, University of East Anglia; Norwich, UK.
[10]School of Health and Life Sciences, Pontifical Catholic University of Rio Grande do Sul; Porto Alegre, 90619-900, Brazil.
[11]School of Life Sciences, University of Nevada Las Vegas; Las Vegas, NV 89154, USA.
[12]Biodiscovery Institute, University of Nottingham; Nottingham, UK.
[13]Department of Immunology, Genetics and Pathology, Science for Life Laboratory, Uppsala University; Uppsala, 751 85, Sweden.
[14]Department of Biological Sciences, Texas Tech University; Lubbock, TX 79409, USA.
[15]Division of Vertebrate Zoology, American Museum of Natural History; New York, NY 10024, USA.
[16]Keck School of Medicine, University of Southern California; Los Angeles, CA 90033, USA.
[17]Fauna Bio Incorporated; Emeryville, CA 94608, USA.
[18]Baskin School of Engineering, University of California Santa Cruz; Santa Cruz, CA 95064, USA.
[19]Faculty of Biosciences, Goethe-University; 60438 Frankfurt, Germany.
[20]LOEWE Centre for Translational Biodiversity Genomics; 60325 Frankfurt, Germany.
[21]Senckenberg Research Institute; 60325 Frankfurt, Germany.
[22]Institute for Systems Biology; Seattle, WA 98109, USA.
[23]School of Biology and Environmental Science, University College Dublin; Belfield, Dublin 4, Ireland.
[24]Department of Experimental and Health Sciences, Institute of Evolutionary Biology (UPF-CSIC),

Universitat Pompeu Fabra; Barcelona, 08003, Spain.
[25]Department of Computational Biology, School of Computer Science, Carnegie Mellon University; Pittsburgh, PA 15213, USA.
[26]Neuroscience Institute, Carnegie Mellon University; Pittsburgh, PA 15213, USA.
[27]Program in Molecular Medicine, UMass Chan Medical School; Worcester, MA 01605, USA.
[28]Department of Epidemiology & Biostatistics, University of California San Francisco; San Francisco, CA 94158, USA.
[29]Gladstone Institutes; San Francisco, CA 94158, USA.
[30]Center for Species Survival, Smithsonian's National Zoo and Conservation Biology Institute; Washington, DC 20008, USA.
[31]Computer Technologies Laboratory, ITMO University; St. Petersburg 197101, Russia.
[32]Smithsonian-Mason School of Conservation, George Mason University; Front Royal, VA 22630, USA.
[33]Department of Biological Sciences, Mellon College of Science, Carnegie Mellon University; Pittsburgh, PA 15213, USA.
[34]Senckenberg Research Institute and Natural History Museum Frankfurt; 60325 Frankfurt am Main, Germany.
[35]Department of Evolution and Ecology, University of California Davis; Davis, CA 95616, USA.
[36]John Muir Institute for the Environment, University of California Davis; Davis, CA 95616, USA.
[37]Morningside Graduate School of Biomedical Sciences, UMass Chan Medical School; Worcester, MA 01605, USA.
[38]Department of Genetics, Yale School of Medicine; New Haven, CT 06510, USA.
[39]Catalan Institution of Research and Advanced Studies (ICREA); Barcelona, 08010, Spain.
[40]CNAG-CRG, Centre for Genomic Regulation, Barcelona Institute of Science and Technology (BIST); Barcelona, 08036, Spain.
[41]Department of Medicine and LIfe Sciences, Institute of Evolutionary Biology (UPF-CSIC), Universitat Pompeu Fabra; Barcelona, 08003, Spain.
[42]Institut Català de Paleontologia Miquel Crusafont, Universitat Autònoma de Barcelona; 08193, Cerdanyola del Vallès, Barcelona, Spain.
[43]Institute of Cell Biology, University of Bern; 3012, Bern, Switzerland.
[44]Department of Biological Sciences, Lehigh University; Bethlehem, PA 18015, USA.
[45]BarcelonaBeta Brain Research Center, Pasqual Maragall Foundation; Barcelona, 08005, Spain.
[46]CRG, Centre for Genomic Regulation, Barcelona Institute of Science and Technology (BIST); Barcelona, 08003, Spain.
[47]Department of Comprehensive Care, School of Dental Medicine, Case Western Reserve University; Cleveland, OH 44106, USA.
[48]Department of Vertebrate Zoology, Canadian Museum of Nature; Ottawa, Ontario K2P 2R1, Canada.
[49]Department of Vertebrate Zoology, Smithsonian Institution; Washington, DC 20002, USA.
[50]Narwhal Genome Initiative, Department of Restorative Dentistry and Biomaterials Sciences, Harvard School of Dental Medicine; Boston, MA 02115, USA.
[51]Department of Evolutionary Ecology, Leibniz Institute for Zoo and Wildlife Research; 10315 Berlin, Germany.
[52]Medical Scientist Training Program, University of Pittsburgh School of Medicine; Pittsburgh, PA 15261, USA.
[53]Chan Zuckerberg Biohub; San Francisco, CA 94158, USA.
[54]Division of Messel Research and Mammalogy, Senckenberg Research Institute and Natural History Museum Frankfurt; 60325 Frankfurt am Main, Germany.
[55]Conservation Genetics, San Diego Zoo Wildlife Alliance; Escondido, CA 92027, USA.
[56]Department of Evolution, Behavior and Ecology, School of Biological Sciences, University of California San Diego; La Jolla, CA 92039, USA.
[57]Department of Organismic and Evolutionary Biology, Harvard University; Cambridge, MA 02138, USA.
[58]Howard Hughes Medical Institute; Chevy Chase, MD, USA.
[59]Department of Ecology and Evolutionary Biology, University of California Santa Cruz; Santa Cruz, CA 95064, USA.

[60]Howard Hughes Medical Institute, University of California Santa Cruz; Santa Cruz, CA 95064, USA.
[61]Department of Evolution, Ecology and Organismal Biology, University of California Riverside; Riverside, CA 92521, USA.
[62]Department of Genetics, University of North Carolina Medical School; Chapel Hill, NC 27599, USA.
[63]Department of Medical Epidemiology and Biostatistics, Karolinska Institutet; Stockholm, Sweden.
[64]Iris Data Solutions, LLC; Orono, ME 04473, USA.
[65]Museum of Zoology, Senckenberg Natural History Collections Dresden; 01109 Dresden, Germany.
[66]Allen Institute for Brain Science; Seattle, WA 98109, USA
